# Identification of Key Diagnostic Markers and Immune Infiltration in Osteoarthritis

**DOI:** 10.2174/1386207325666220426083526

**Published:** 2023-01-05

**Authors:** Mingyue Yan, Haibo Zhao, Zewen Sun, Jinli Chen, Yi Zhang, Jiake Gao, Tengbo Yu

**Affiliations:** 1 Department of Orthopaedics, The Affiliated Hospital of Qingdao University, Qingdao, Shandong 266500, China

**Keywords:** Osteoarthritis, GEO, immune cells, immune infiltration, bioinformatics, ITGAM

## Abstract

***Background*:** Osteoarthritis (OA) is a worldwide chronic disease of the articulating joints. An increasing body of data demonstrates the immune system's involvement in osteoarthritis. The molecular mechanisms of OA are still unclear. This study aimed to search for OA immune-related hub genes and determine appropriate diagnostic markers to help the detection and treatment of the disease.

***Methods*:** Gene expression data were downloaded from the GEO database. Firstly, we analyzed and identified the differentially expressed genes(DEGs)using R packages. Meanwhile, ssGSEA was used to determine the activation degree of immune-related genes (IRGs), and WGCNA analysis was applied to search for co-expressed gene modules associated with immune cells. Then, critical networks and hub genes were found in the PPI network. Gene Ontology (GO) annotation and Kyoto Encyclopedia of Gene and Genomes (KEGG) pathway enrichment analyzed the biological functions of genes. The ability of the hub genes to differentiate OA from controls was assessed by the area under the ROC curve. A miRNA and transcription factor (TF) regulatory network was constructed according to their relationship with hub genes. Finally, the validation of hub genes was carried out by qPCR.

***Results*:** In total, 353 DEGs were identified in OA patients compared with controls, including 222 upregulated and 131 downregulated genes. WGCNA successfully identified 34 main functional modules involved in the pathogenesis of OA. The most crucial functional module involved in OA included 89 genes. 19 immune-related genes were obtained by overlapping DEGs with the darkgrey module. The String database was constructed using the protein-protein interaction (PPI) network of 19 target genes, and 7 hub genes were identified by MCODE. ROC curve showed that 7 hub genes were potential biomarkers of OA. The expression levels of hub genes were validated by qPCR, and the results were consistent with those from bioinformatic analyses.

***Conclusion*:** Immune-related hub genes, including TYROBP, ITGAM, ITGB2, C1QC, MARCO, C1QB, and TLR8, may play critical roles in OA development. ITGAM had the highest correction on immune cells.

## 1 INTRODUCTION

Osteoarthritis (OA) is a highly prevalent, age-dependent joint disease, particularly among older people with multiple comorbid illnesses [1]. It is regarded as a multifactorial disease, with common risk factors including age, excess, mechanical loading, and metabolic syndrome [2]. Bone destruction, synovial hyperplasia, osteophyte formation and subchondral bone sclerosis are the main features of osteoarthritis, and the typical symptom is severe joint pain [3]. Specifically, knee osteoarthritis is a painful disease and substantially decreases the life quality of suffered patients [4]. Consequent to unprecedented global population aging, osteoarthritis remains a leading cause of disability, and the treatment often comes at a high cost [5], imposing an enormous economic burden worldwide. Unfortunately, few effective disease-modifying therapies are available due to the unknown etiology and pathological mechanisms. As a consequence, identifying new and effective treatments is vital to improve osteoarthritis outcomes and guide clinical management.

Cumulating evidence supports the pathological roles of immune and inflammatory in the occurrence and development of osteoarthritis [6, 7]. Some studies proposed that altered gene expression in chondrocytes osteoarthritis progression by regulating immune responses [8, 9]. Pro-inflammatory cytokines play a central role in chondrocyte apoptosis and cartilage matrix proteolysis and thus may be implicated in the pathogenesis of osteoarthritis [10]. Previous studies showed that the extent of immune dysregulation significantly correlates with the severity of osteoarthritis, revealing an association between immunity response and clinical manifestations [11]. Therefore, exploring immune infiltration and related diagnostic gene markers in OA will provide a deeper understanding of the pathogenesis of osteoarthritis.

In this study, with the aid of Gene Expression Omnibus (GEO) and bioinformatics, we aimed to describe the characteristics of infiltrating immune cells in osteoarthritis tissues, and comprehensive bioinformatics analyses were performed to explore underlying hub genes. Besides, we constructed a gene-TF-miRNA co-regulatory network according to the relationship among hub genes, TF, and miRNA by bioinformatics methods.

## 2 MATERIALS AND METHODS

### 2.1 Data Source

The RNA expression datasets of OA were obtained from Gene Expression Omnibus (GEO) database (https://www.ncbi.nlm.nih.gov/geo/). The gene expression profiles of human knee osteoarthritis tissues were downloaded from GSE114007 (20 OA patients and 18 control individuals) and GSE143514 (5 OA patients and 3 control individuals) for training (Training Set), respectively. The gene expression profiles for validation (Validation Set) were downloaded from GSE98918 (12 OA patients and 12 control individuals).

### 2.2 Acquirement of Differentially Expressed Genes (DEGs)

Generally, all microarray data after normalization were analyzed by R software. After removing interbatch differences, R package “DESeq2” was used to identify differentially expressed mRNAs between OA and control samples with ∣log2FC| >1 and adj.P.Val < 0.05 as the threshold. The DEGs' heatmap cluster and volcano plots were created using the “heatmap” and “ggplots” packages *via* R software.

### 2.3 Functional Annotation and Pathway Enrichment Analysis

GO annotation and KEGG enrichment analysis were performed using the “clusterProfiler” package to reveal the functions of DEGs. The GO functions include biological processes, cellular components, and molecular functions. The KEGG pathway analysis was prone to describe gene function at the genomic and molecular levels and show the correlated genes. *P* < 0.05 was considered statistically significant.

### 2.4 Infiltration of Immune Cells

The GEO gene expression profile data were used to quantify the infiltration of immune cells in knee tissues by ssGSEA (Single-sample gene set enrichment analysis), and the infiltration of 28 immune cells was obtained. Each resulting ssGSEA score represents the degree to which the genes in a particular gene set are co-ordinately up or downregulated in each sample in the dataset. The ssGSEA ranked the genes by their absolute expression in a sample, and the enrichment score was calculated by an integration of the differences between the empirical cumulative distribution functions. Furthermore, we performed Spearman correlation analysis on infiltrating immune cells and gene expression by the “ggpubr” package.

### 2.5 WGCNA

The WGCNAR package was used for weighted gene co-expression network analysis on the GEO expression file. Firstly, samples were clustered to assess the presence of any outliers. Then, the automatic network construction function was used to get the co-expression network. The pickSoftThreshold was used to calculate the soft thresholding power β. Furthermore, hierarchical clustering and the dynamic tree cut function were used to detect different modules. Finally, the Pearson correlation coefficient between the sample vector of variables and the characteristic gene of the module was calculated to measure the degree of association between the clinical features and the modules. We then found out which module of immune cell genes was the most relevant in OA.

### 2.6 Protein-protein Interaction (PPI) Network Construction

Analysis of the functional interactions between proteins could provide essential insights into the progression of OA. We constructed the PPI network by STRING database (https://string-db.org/). A combined score of >0.4 was considered significant. Cytoscape performed the visualized PPI network. MCODE was used to screen the significant modules in the PPI network. The genes with the highest degree of connectivity in the module were considered hub genes. They were expected to exhibit higher biological significance than the other gene members of the module.

### 2.7 Validation of Hub Genes

The expression levels of hub genes were uesd to verify whether the hub genes were essential to OA. The “*pROC”* package was used to perform ROC curve analysis to evaluate the diagnostic value of hub genes for OA, and the area under the curve (AUC) was then computed. The higher the AUC value was, the better the ability of the gene to distinguish whether the sample was diseased or not. The genes with AUC > 0.7 were considered diagnostic genes of OA.

### 2.8 Correlation Analysis between Hub Genes and Infiltrating Immune Cells

The “ggpubr” package was used to perform Spearman correlation analysis on hub genes and infiltrating immune cells,and the “ggplot2” package was used to visualize the results.

### 2.9 Multi-factor Regulation Network Construction

We used NetworkAnalyst (https://www.networkanalyst.ca/) and miRNet databases (https://www.mirnet.ca/) to predict the TFs and miRNAs of hub genes. The data of hub genes and their TFs and miRNAs were integrated into a regulatory network and visualized using Cytoscape software.

### 2.10 RT-PCR Validation of the Hub Genes

To confirm the results of bioinformatics analysis, cartilage tissues of OA patients undergoing knee replacement and normal patients were collected for RT-PCR validation. The study protocol was approved by the Ethics Committee of The Affiliated Hospital of Qingdao University (approval number: QYFY WZLL 26788), and all patients signed the informed consent for tissue collection.

Total RNA of cartilage tissue was extracted with TRIzol reagent (Invitrogen, USA). Those RNA samples were reverse transcribed to cDNA with the PrimeScript RT reagent Kit and subsequently amplified using the TB Green Premix Ex Taq II kit (TaKaRa, Japan). Primer sequences of mRNA were shown in Table **1**. The primers of all mRNAs were purchased from ThermoFisher (USA). The gene expression was normalized for GAPDH levels and calculated using the 2^−ΔΔCt^ method. P < 0.05 indicated a significant difference.

## 3 RESULTS

### 3.1 Identification of Differentially Expressed Genes (DEGs)

**Fig. (1):**
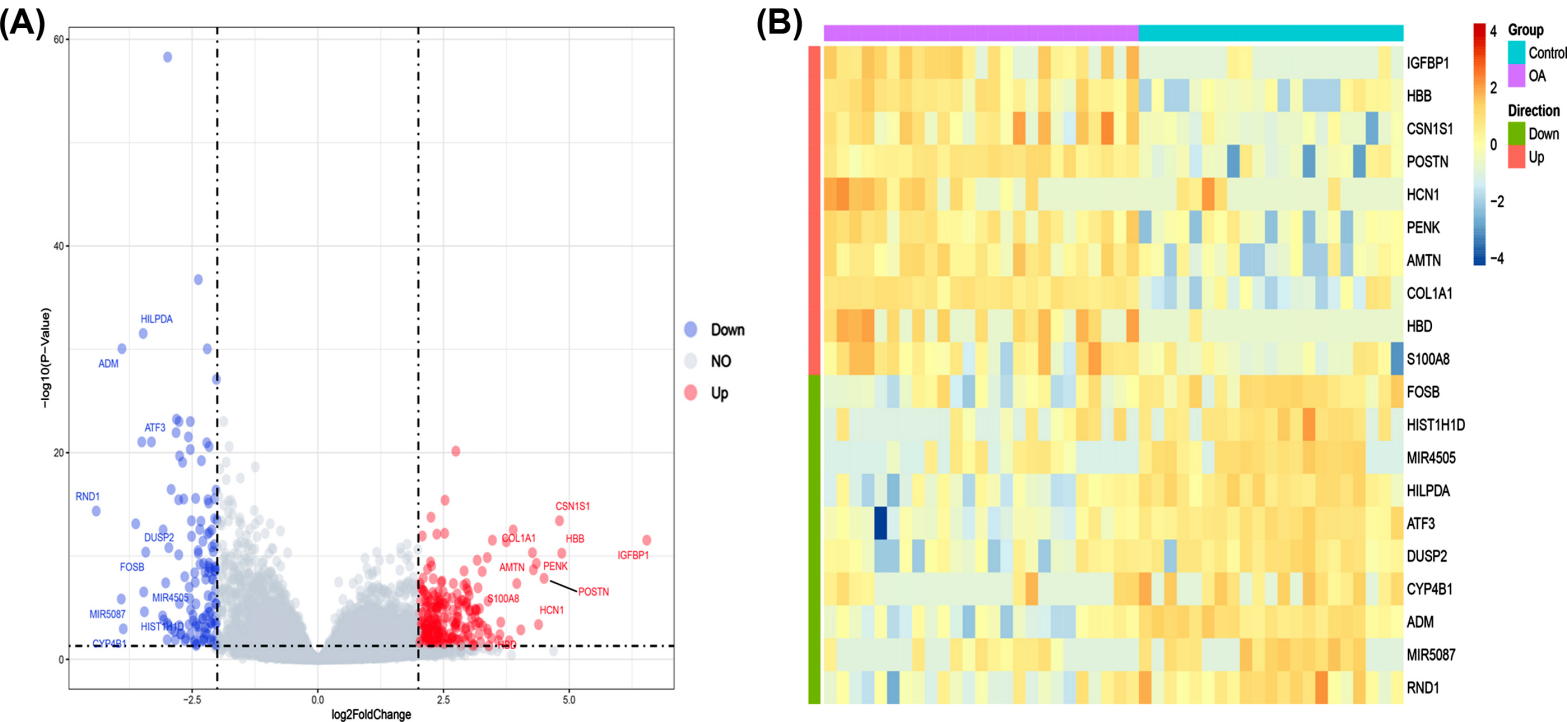
Identification of differentially expressed genes (DEGs) in osteoarthritis (OA). (**A**) Volcano plot of all DEGs in OA. (**B**) Heat map of the top 10 upregulated and downregulated DEGs in OA.

First, the batch effect was removed from the gene expression matrix after merging the GSE114007 and GSE143514 datasets. Then we used the “DESeq2” package to extract a total of 353 DEGs (Supplementary File **1**), including 222 downregulated genes and 131 upregulated genes, which were shown in volcano plots (Fig. **1A**). The top 10 upregulated and downregulated DEGs in the visualized heatmap were sorted by the log2FC of DEGs (Fig. **1B**).

### 3.2 GO Annotation and KEGG Pathway Enrichment Analysis of the DEGs

**Fig (2):**
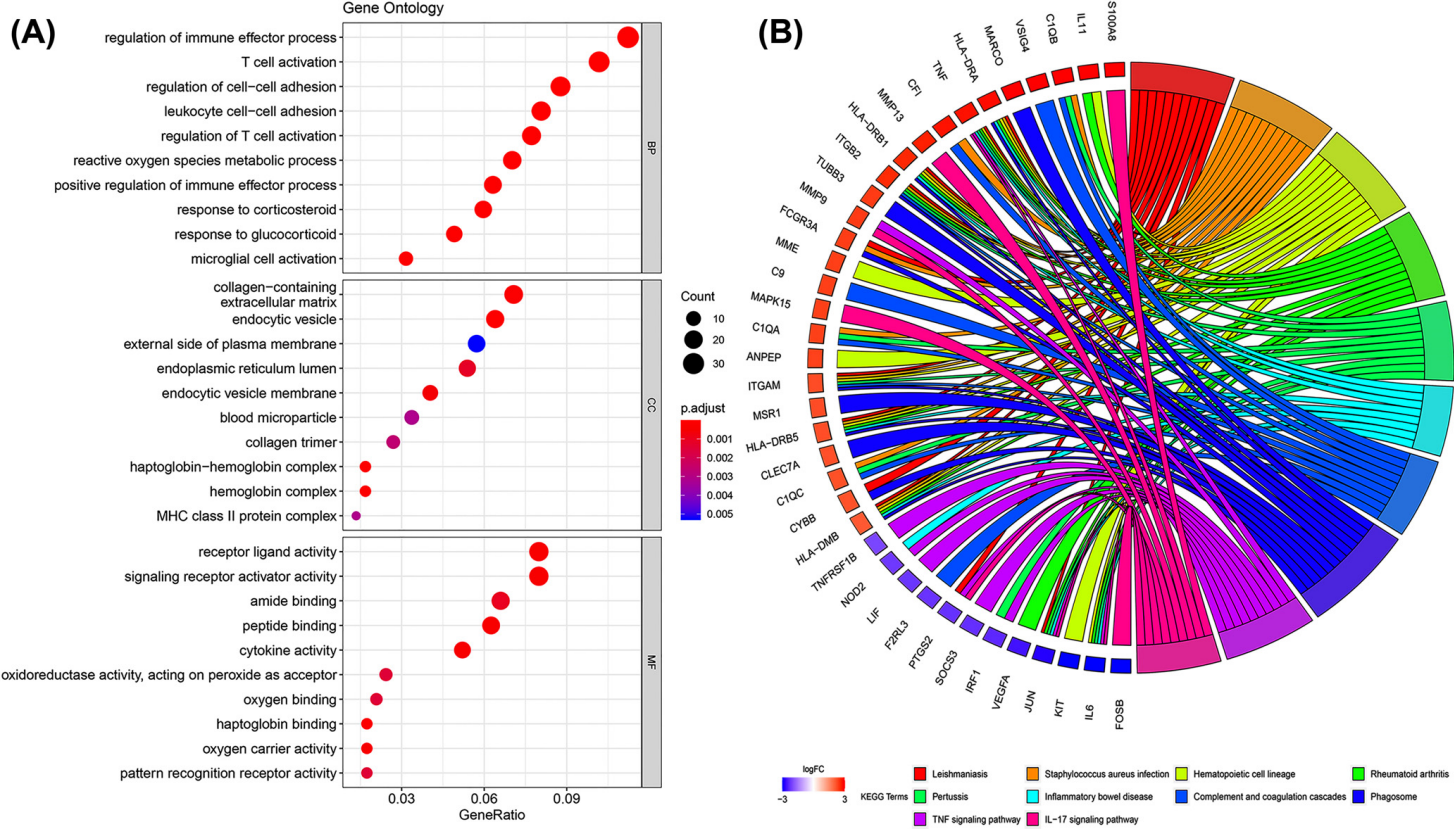
Functional enrichment analysis of DEGs. (**A**) GO enrichment of DEGs in GSE114007 and GSE143514 data. GO analysis included 3 functional groups: molecular functions, biological processes, and cell components. (**B**) KEGG of DEGs in GSE114007 and GSE143514 data.

To obtain the functions of these 353 DEGs, GO annotation and KEGG enrichment analysis were conducted using the “clusterProfiler” package. For GO biological process, the DEGs were mainly enriched in regulating the immune effector process, T cell activation, and cell−cell adhesion. In the cellular component, the DEGs were mainly enriched in the collagen-containing extracellular matrix, endocytic vesicles, and the external side of the plasma membrane. The top three significantly enriched terms were receptor ligand activity, signaling receptor activator activity, and amide binding in the molecular function group (Fig. **2A**). Besides, the top ten GO terms in each group are listed in Table **2**. The result of the KEGG pathway enrichment analysis is shown in Fig. (**2B**). Leishmaniasis, Staphylococcus aureus infection, and Hematopoietic cell lineage were highly associated with OA. The above results indicated that the DEGs were mainly related to immune-related functions. Additionally, the top ten pathways are listed in Table **3**.

### 3.3 Immune Infiltration Analyses

**Fig. (3):**
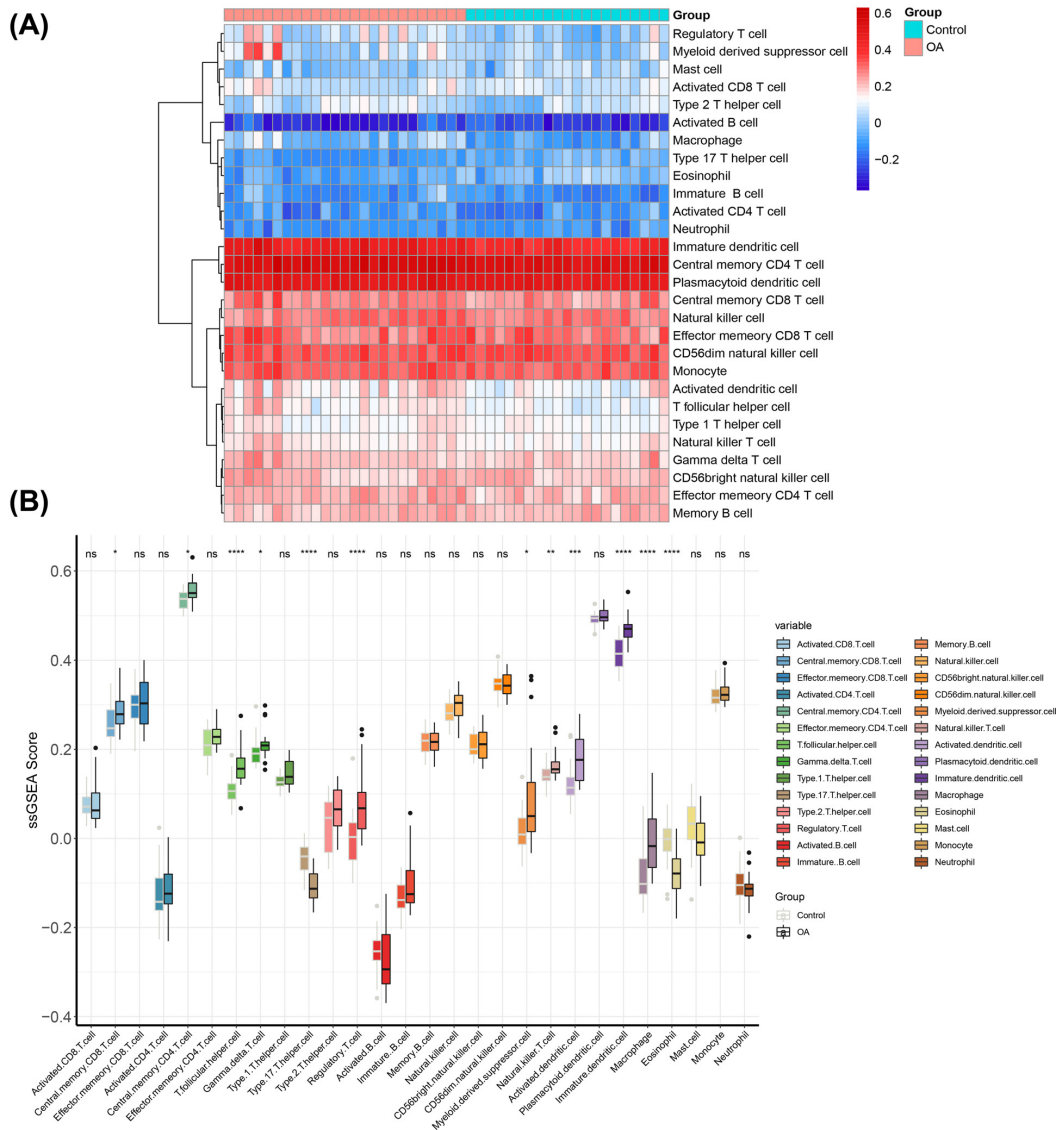
The landscape of immune infiltration between OA and controls. (**A**) The heat map summarizes the immune infiltration score between OA and controls. (**B**) The difference in immune infiltration between OA and controls. (*P*-value < 0.05 indicated statistical significance).

The difference in the infiltration of 28 immune cells between OA and control was investigated by ssGSEA. The scores of the infiltration of immune cells between OA patients and control were summarized by a heatmap (Fig. **3A**). The boxplot showed that 12 immune cells had higher infiltration rates in OA samples (Fig. **3B**, *P* < 0.05).

### 3.4 Weighted Co-expression Network Construction and Identification of Key Modules

**Fig (4):**
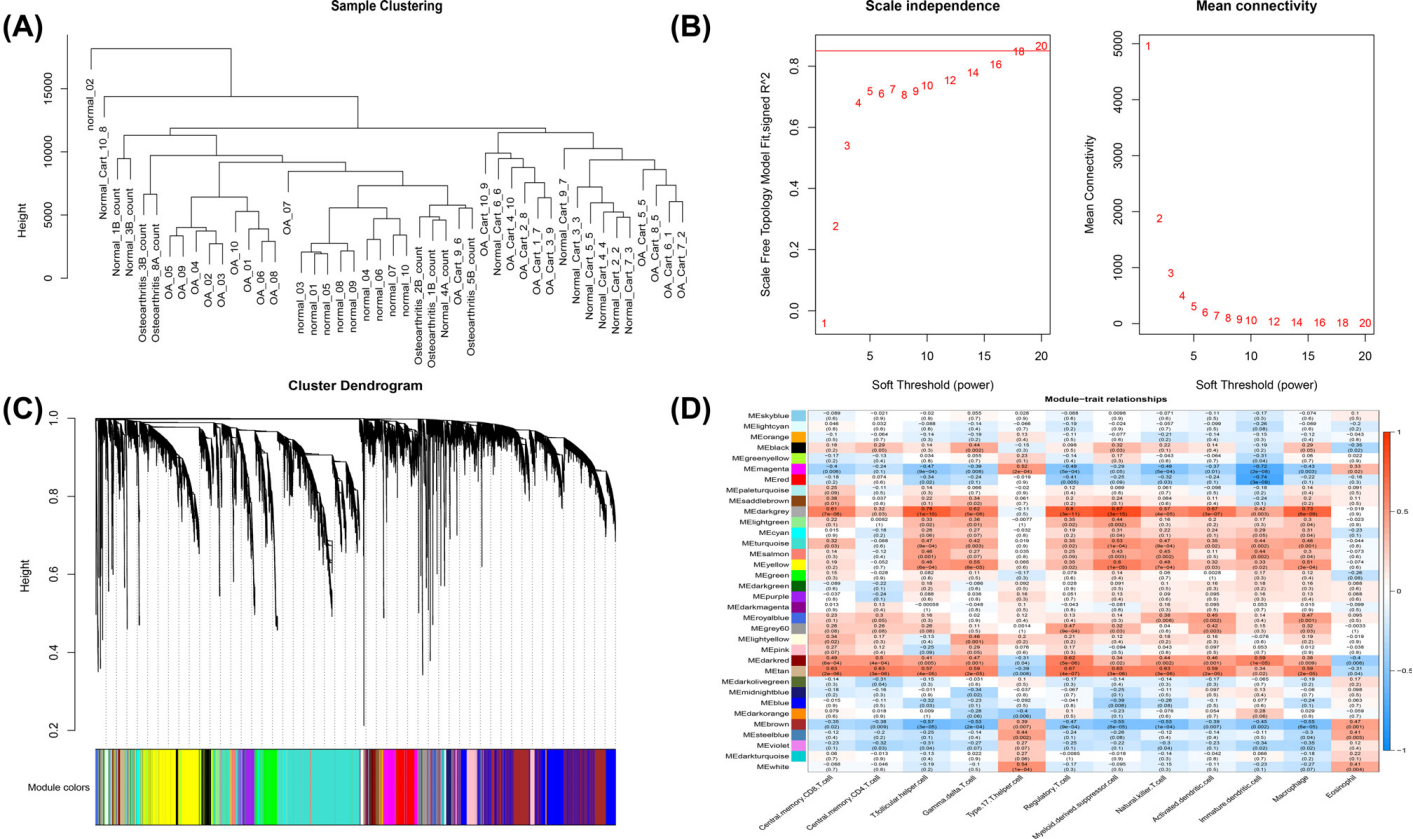
Identification of key modules correlated with immune cells through WGCNA. (**A**) Clustering dendrograms of samples. (**B**) Analysis of network topologies for various soft‐thresholding powers through the scale‐free fit index (left) and mean connectivity (right). (**C**) Clustering dendrogram of genes based on topological overlapping. Different colors were assigned to the corresponding modules. A total of 34 modules were identified. (**D**) Heatmap of the correlation between module eigengenes and immune cells.

Pearson’s correlation coefficient was used to cluster the samples. There was no outlier to remove (Fig. **4A**). The soft threshold was set to 18 to construct a scale-free network (Fig. **4B**). 34 modules were then identified based on average hierarchical clustering and dynamic tree clipping (Fig. **4C**). The darkgrey module was highly related to 12 immune cells. Thus, this module, including 89 genes, was selected for further analysis (Fig. **4D**).

### 3.5 GO and KEGG Enrichment Analysis of Immune-related Genes

**Fig. (5):**
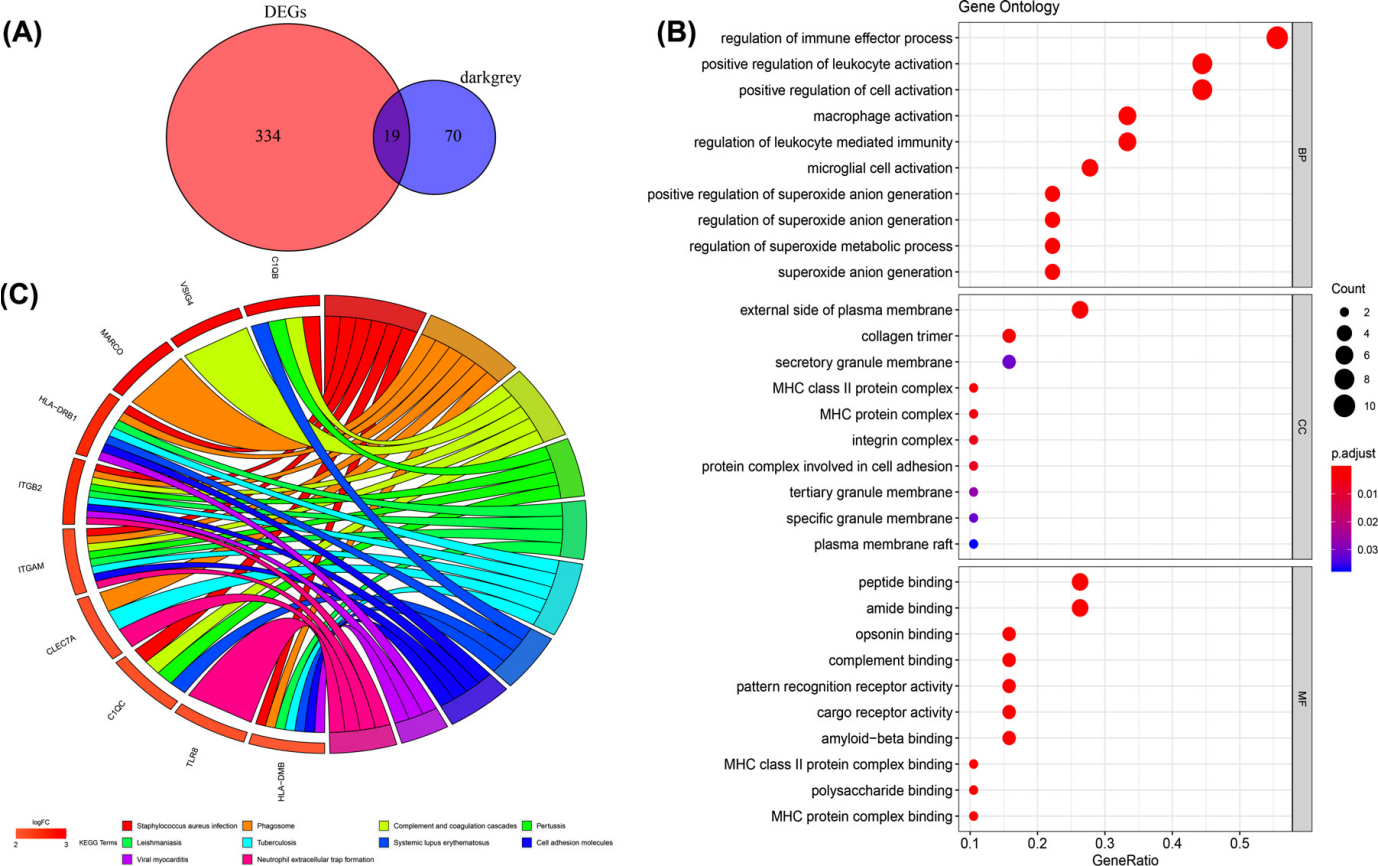
Venn diagram and functional enrichment analysis of immune-related genes. (**A**) Venn diagram of immune-related genes extracted from DEGs and darkgery modules. (**B**) GO enrichment of immune-related genes in GSE114007 and GSE143514 data. (**C**) KEGG of immune-related genes in GSE114007 and GSE143514 data.

Darkgrey module genes and DEGs were overlapped to obtain 19 immune-related genes (Fig. **5A**). Then GO analysis and KEGG analysis of the immune-related genes were conducted. The results showed that the related genes were mainly associated with biological processes such as regulation of immune effector process, positive regulation of leukocyte activation, macrophage activation, and T cell activation. As for GO-CC, the genes were enriched in the external side of the plasma membrane, secretory granule membrane, and MHC protein complex. Meanwhile, regarding GO-MF, the genes were mainly enriched in peptide binding, complement binding, and pattern recognition receptor activity (Fig. **5B**). Besides, the top ten GO terms in each group are listed in Table **2** . Finally, the KEGG analysis revealed that the genes were significantly involved in the phagosome, neutrophil extracellular trap formation, and complement and coagulation cascades (Fig. **5C**). Additionally, the top ten pathways are listed in Table **3**.

### 3.6 PPI Network Construction and Module Analysis

**Fig. (6):**
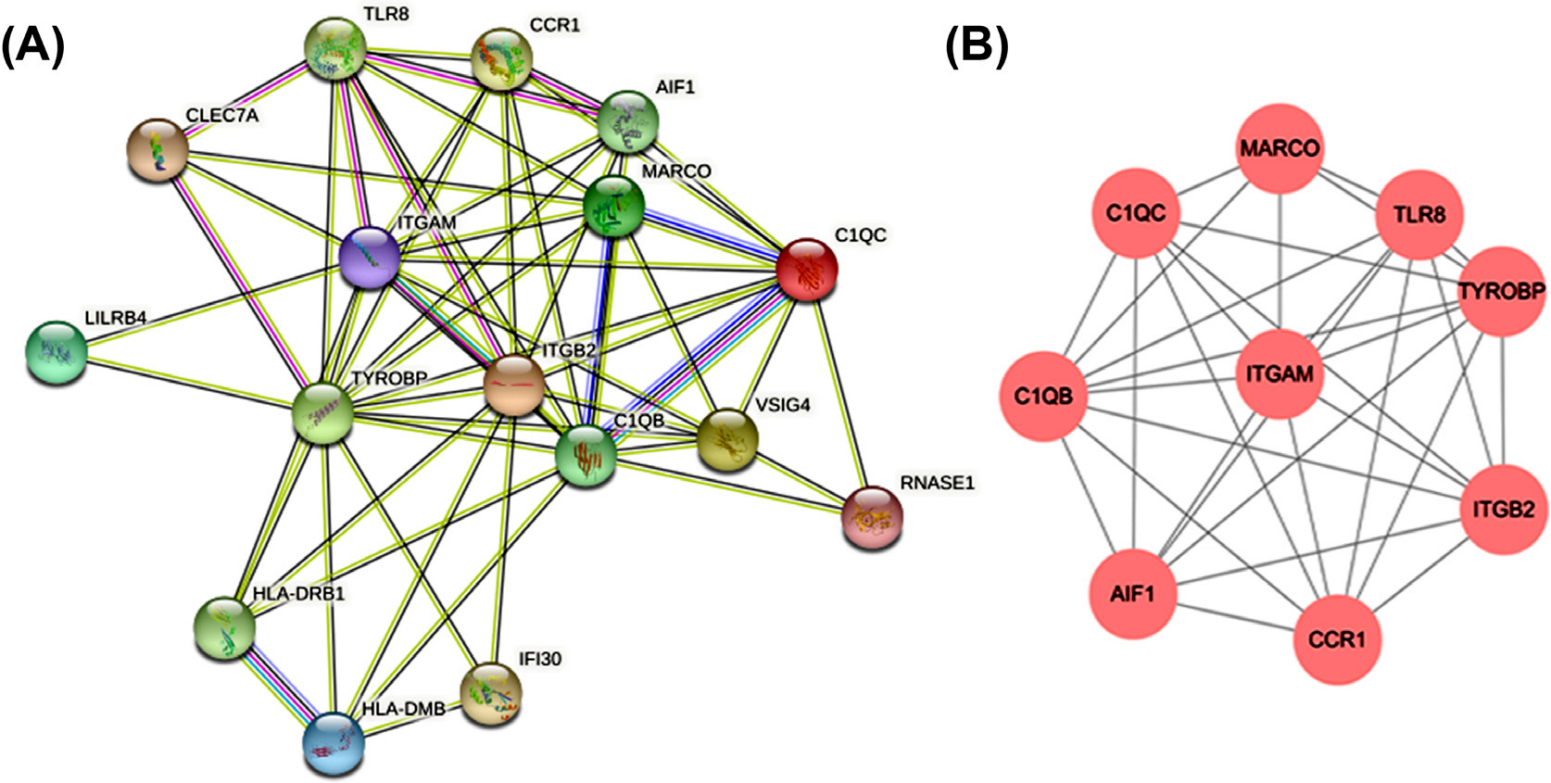
Construction of PPI network, analysis of key modules, and identification of hub genes. (**A**) The whole PPI network. (**B**) PPI network of hub genes.

To further study the interaction of 19 immune-related genes, the PPI network was constructed using by STRING database. With the confidence >0.4 and hiding the disconnected nodes, a visualized PPI network was created by Cytoscape (Fig. **6A**). 9 genes in the key module were selected as hub genes using the MCODE plugin (Fig. **6B**).

### 3.7 Hub Gene Validation

**Fig (7):**
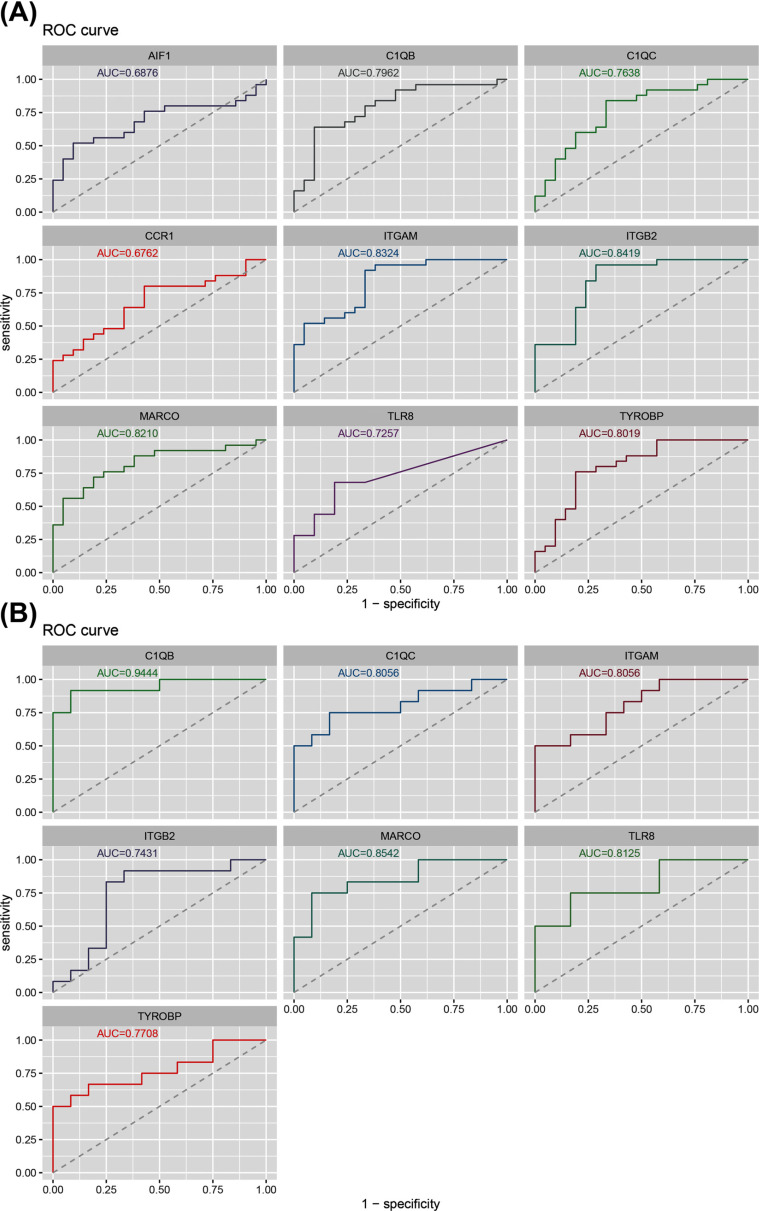
ROC curves for evaluating the accuracy of logistic regression analysis of hub genes. (**A**) GSE114007 and GSE143514. (**B**) GSE98918.

A Receiver Operating Characteristic (ROC) curve was plotted, and the area under the curve (AUC) was calculated to evaluate the diagnostic value of hub genes. The AUCS of 7 hub genes, including TYROBP, ITGAM, ITGB2, C1QC, MARCO, C1QB, and TLR8, were all greater than 0.7 (Fig. **7A**). Therefore, these hub genes have a higher diagnostic value as a biomarker.

Furthermore, the expression levels of the 7 hub genes were examined in GSE98918. The AUC of 7 hub genes was also greater than 0.7 (Fig. **7B**). It has been determined that these hub genes could be used as diagnostic markers of OA.

### 3.8 Correlation Analysis between Hub Genes and Infiltrating Immune Cells

**Fig (8):**
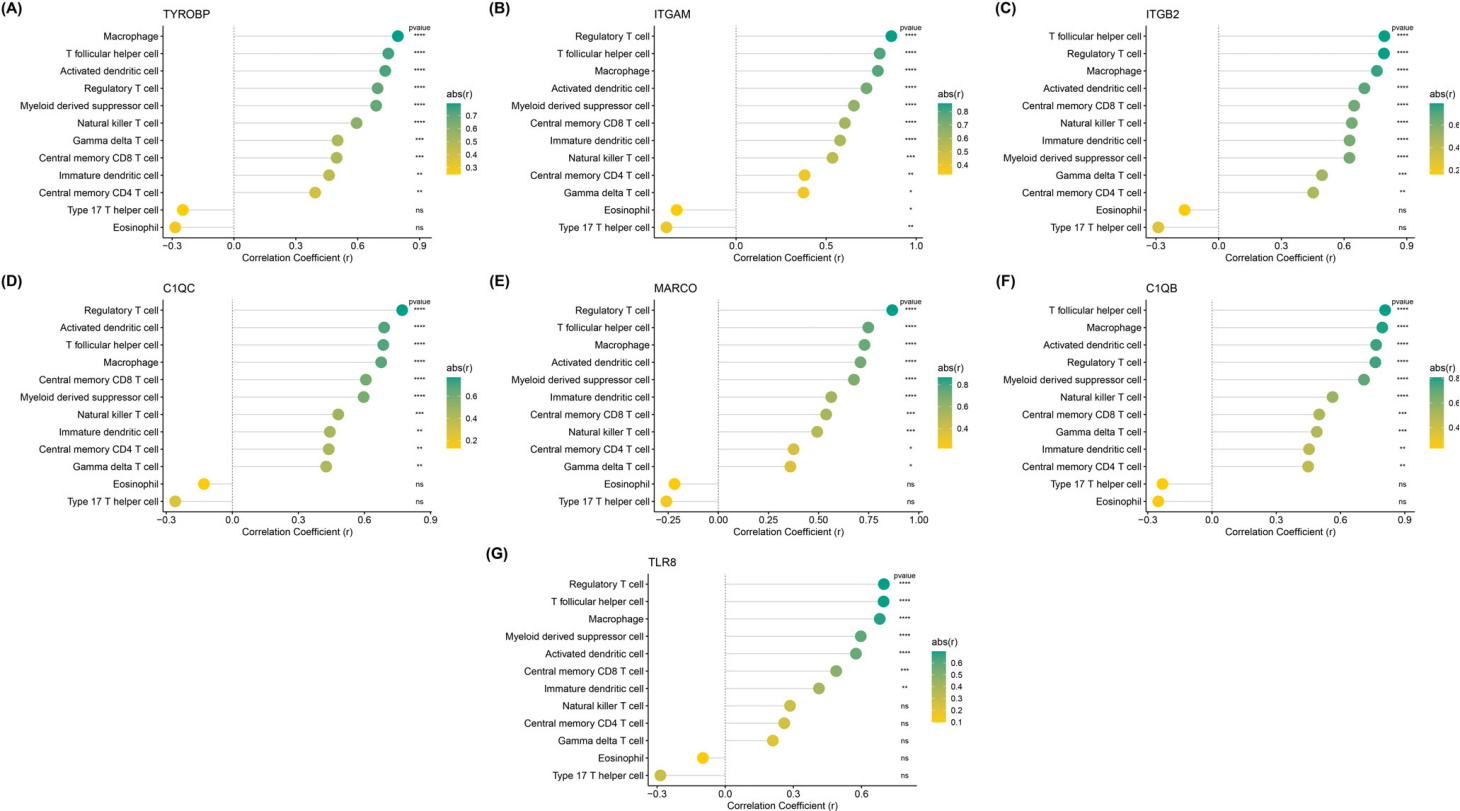
Association between hub genes and immune cell infiltration.

Exploring the relationship between hub genes and immune cells indicated that ITGAM had the highest correction with immune cells. ITGAM was negatively correlated with Type 17 T helper cells and Eosinophils but positively correlated with other immune cells (Fig. **8**).

### 3.9 Multi-factor Regulation Network Construction

**Fig (9):**
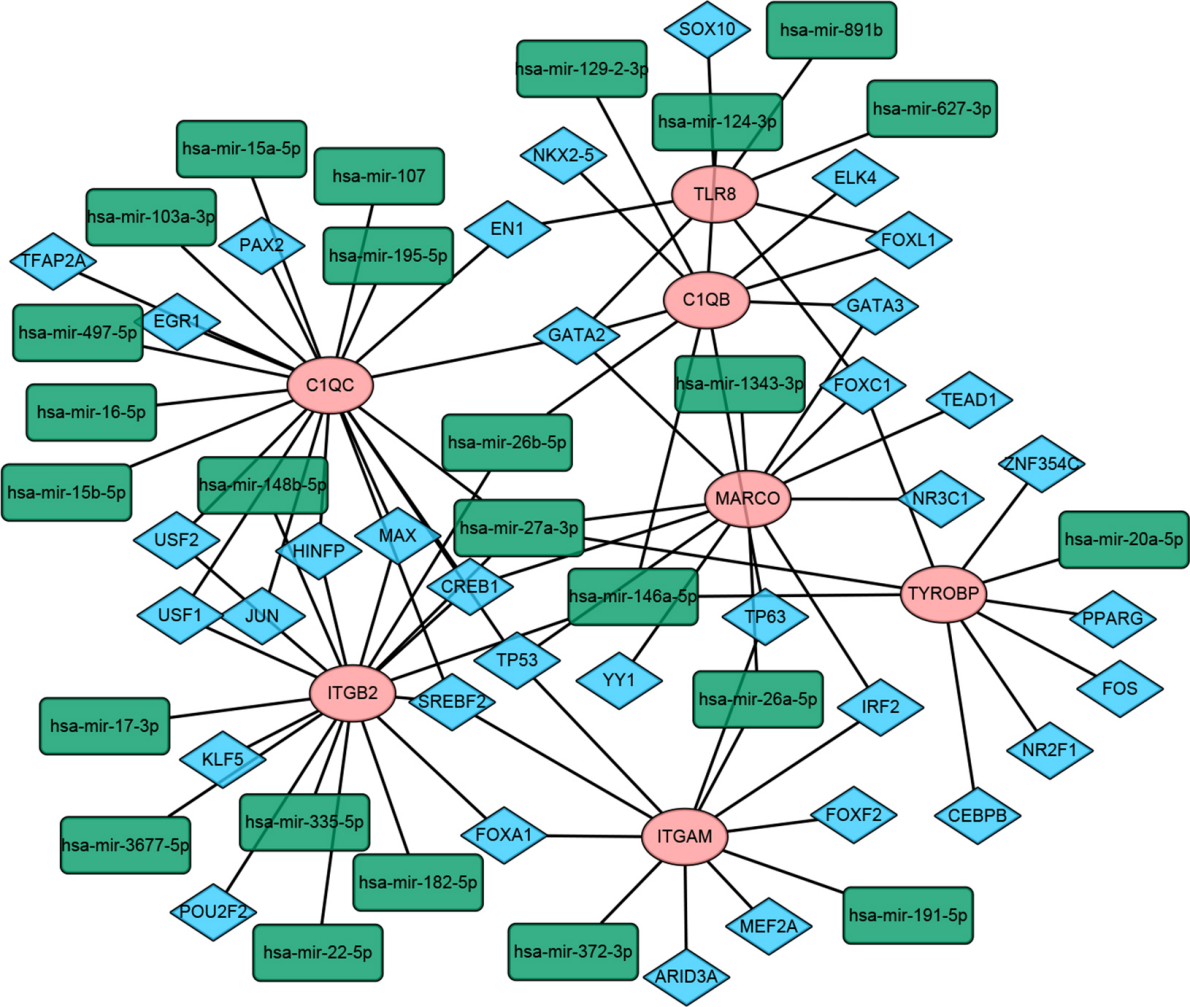
Construction of the hub gene-TF-miRNA network in OA.

Based on miRNet and NetworkAnalyst databases, the miRnas hub genes and TFs-hub gene networks were constructed with the help of Cytoscape software (Fig. **9**). The network was composed of 7 hub genes, 25 miRNAs, and 36 TFs.

The hub gene-TF-miRNA network consisted of 7 hub genes (pink ellipse nodes), 25 miRNAs (green rectangle nodes), and 36 TFs (blue diamond nodes).

### 3.10 RT-PCR Validation of the Hub Genes

**Fig. (10):**
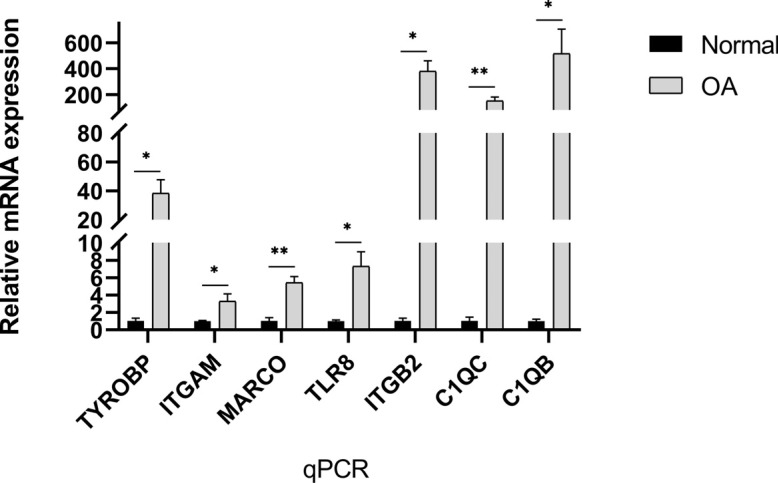
RT-PCR validation of the hub gene between OA and controls. Each experiment was repeated in triplicate, and the results were presented as M ± SD. (∗*p* < 0.01, ∗∗*p* < 0.001).

Quantitative PCR (qPCR) analyses were performed and showed the relative expression levels of 7 hub genes, including TYROBP, ITGAM, ITGB2, C1QC, MARCO, C1QB and TLR8 (Fig. **10**). The results were consistent with those from bioinformatic analyses.

## 4 DISCUSSION

Osteoarthritis (OA), especially knee OA, is a chronic disease of the articulating joints, followed by activation of the inflammatory response involving the interaction of cartilage, subchondral bone, and synovium [12], causing joint pain, stiffness, and functional disability in patients worldwide. Previous studies have identified immune cells as significant regulators in the metabolic activities of chondrocytes [12-14]. Therefore, finding immune-related hub genes and diagnostic gene markers to help detect and treat the disease. The present study was conducted to identify 353 DEGs and 19 immune-related genes. A total of 7 hub genes were identified based on the integrated analysis of the PPI network, and ITGAM was indicated to have the highest correction for immune cells. The following analysis also revealed a hub-gene-related TF-miRNA network.

To understand the functions of 353 DEGs, GO annotation and KEGG enrichment analysis were conducted, and found that these genes were associated with immune-related processes. WGCNA revealed 19 immune-related genes, and following enrichment analysis showed that they were mainly associated with biological processes, such as the regulation of the immune effector process, positive regulation of leukocyte activation, macrophage activation, and T cell activation. It was reported that neutrophil elastase released by activated leukocytes could activate MMP-13, PAR2, and a series of cascade reactions, leading to severe cartilage destruction [15, 16]. It was proved that osteophyte formation was related to PAR2 [17, 18]. Suppression of PAR2 would inhibit the apoptosis and senescence of chondrocytes by activating autophagy [19]. Besides, increased macrophage infiltration in the synovium of OA could upregulate the production of anti-inflammatory cytokines such as IL-1 and TNF-α [20, 21]. M1 polarization was associated with the release of R-spondin-2 and the activation of β-catenin signaling, which could exacerbate the progression of osteoarthritis [22]. However, M2 macrophages could inhibit the hypertrophy and apoptosis of chondrocytes, protecting cartilage and promoting cartilage repair [23]. It was also revealed that activated T helper cells would induce osteoclast formation and promote the progression of osteoarthritis by inducing macrophage inflammatory protein-1γ [24]. These genes were also involved in molecular functions, for instance, complement binding and pattern recognition receptor activity. It was found that complement factors, produced and activated in OA joints, suggested an association between the complement alternative pathway components and joint inflammation, supporting the involvement of innate immunity in OA [25]. Besides, pattern recognition receptors could recognize the cellular debris released during chondrocyte degeneration and apoptosis, activating downstream signal pathways and triggering an intense inflammatory response [26, 27]. On the whole, GO annotation and KEGG enrichment analysis indicated a strong correlation between these genes and immune-related processes related to OA progression.

A PPI network was constructed to further study the interaction of 19 immune-related genes. Then, 7 hub genes (TYROBP、ITGAM、ITGB2、C1QC、MARCO、C1QB、TLR8) were identified with AUC> 0.7, which were recognized as diagnostic biomarkers of OA. Exploring the relationship between hub genes and immune cells indicated that ITGAM had the highest correction with immune cells. ITGAM, a marker for total macrophages, was considered a risk factor for systemic lupus erythematosus and possibly a protective factor to rheumatoid arthritis [28]. Meanwhile, ITGAM has been reported to encode CD11b, prevent chondrocyte mineralization, and alleviate OA progression [29, 30]. Besides, TYROBP, together with OSCAR, was proved to regulate the immunoreceptor tyrosine-based activation motif (ITAM) signaling pathway, which was strongly associated with osteoclastogenesis [31]. A study suggested that ITGB2, one of the integrin family, was significantly increased in OA meniscal compared with the control group, consistent with our previous study [32]. Besides, ITGB2 was involved in the phosphate metabolic process, with the clinical observation that meniscal calcification was more severe in OA menisci [33]. It was revealed that C1QB and C1QC, encoding C1q, were increased under inflammatory stimuli relevant to OA, and binding of C1q to chondrocytes would lead to changes in collagen expression with a decrease in type II collagen [34]. MARCO, an M1 macrophage marker, was reported to be significantly associated with early synovitis and the process of including increased trafficking and migration of monocytes into the synovial membrane [35]. A previous study showed that TLR8 expressing M1 macrophages could exacerbate cartilage degeneration in response to a joint injury [36]. Furthermore, when overexpressing human TLR8 in mice, researchers found that huTLR8 would promote the exacerbation of arthritis, and the levels of huTLR8 were related to proinflammatory cytokines in mice joints [37]. As a whole, these hub genes are valuable for being recognized as diagnostic biomarkers of OA. It has also been observed that several studies reported some diagnostic biomarkers in osteoarthritis, which were different from the hub genes found or derived from the same gene family [38-40]. The possible reasons might be the differences in the resources of the datasets analyzed. In any event, further experimental work will be required to test the value of these markers. Finally, we constructed a gene-TF-miRNA co-regulatory network based on the relationship among hub genes, TF, and miRNA, which provides a resource for future mechanistic studies of OA.

Nevertheless, there were several potential limitations to this study. First, the data were obtained mainly from the GEO database, and immune cell infiltration of OA was not validated experimentally. Second, although we performed quality control of the original data, larger samples should be analyzed to validate the results. Finally, further elucidation of related molecular and validation of mechanisms have to be done to provide new ideas for postponing the development of osteoarthritis [41, 42].

## CONCLUSION

Overall, this study identified 19 immune-related genes and 7 hub genes, and a TF-miRNA network (7 hub genes, 25 miRNAs, and 36 TFs) was successfully built. ITGAM was found to be significantly related to immune cells in OA. Furthermore, the biological functions and pathways of the identified genes provide a more detailed molecular mechanism of immune infiltration in OA development. Related diagnostic gene markers in OA will help to facilitate our understanding of the mechanisms governing osteoarthritis.

## Figures and Tables

**Table 1:** Primer sequences of hub genes and Tm values.

**Prime Name**	**Sequence**	**Tm**
h-GAPDH-F	CATGTTCGTCATGGGTGTGAA	59
h-GAPDH-R	GGCATGGACTGTGGTCATGAG	60
h-TYROBP-F	TTGCAGTTGCTCTACGGTGA	60
h-TYROBP-R	TACGCTGTTTCCGGGTCG	60
h-ITGAM-F	TGCTGCGCCTGAACTTCTCT	60
h-ITGAM-R	GGTCATCCTGGCAGATGTTGT	58
h-ITGB2-F	TGGACCGCTACCTCATCTATGTG	60
h-ITGB2-R	GGTCGCTCAGGTGGATCAGA	60
h-C1QC-F	AGAAGGGAGAACCCGGCTTAC	60
h-C1QC-R	ACCGTGAACACTGACTGGAATTT	59
h-MARCO-F	ATTGTCTTCTGCCGCATGCT	59
h-MARCO-R	GTCATGATGGCCCCAGCTAT	58
h-C1QB-F	TGCCACAAGAACCATCAACGT	60
h-C1QB-R	CTGGCGTGGTAGGTGAAGTAGTAG	58

**Table 2:** Top 10 GO analyses results of DEGs and IRGs sorted according to adjusted *P*-value).

**Group**	**Term**	**Gene**	**Count**	***P*-value**
DEGs	Regulation of immune effector process	C1QA/C1QB/C1QC/C9/CFI/CLEC12B/CLEC7A/CPN2/FOXJ1/HERC5/HLA-DMB/HLA-DRA/HLA-DRB1/HTRA1/IL6/IRF4/ITGAM/ITGB2/LILRB4/MYB/NOD2/PCK1/PLA2G5/RAC2/RARA/SLAMF6/SLC7A5/TNF/TNFRSF1B/TYROBP/VSIG4/WNT5A	32	8.71E-13
	Response to corticosteroid	ADM/AGTR2/ALPL/AVPR1A/CDKN1A/COL1A1/CSN1S1/CYBB/DDIT4/ERRFI1/FOSB/GJB2/HSD11B2/IL6/PCK1/STC1/TNF	17	1.52E-10
	T cell activation	AIF1/CD70/CLEC7A/EGR3/FOXJ1/HES1/HLA-DMB/HLA-DRA/HLA-DRB1/IFNA5/IFNE/IL6/IRF1/IRF4/KIT/LILRB4/MIR27A/MYB/NOD2/PAK3/PAX1/PCK1/PIK3CG/RAC2/RARA/SLAMF6/THY1/TNFRSF1B/VSIG4	29	1.83E-10
	Regulation of T cell activation	AIF1/CD70/EGR3/FOXJ1/HES1/HLA-DMB/HLA-DRA/HLA-DRB1/IL6/IRF1/IRF4/LILRB4/MIR27A/MYB/NOD2/PAK3/PCK1/RAC2/RARA/THY1/TNFRSF1B/VSIG4	22	5.71E-09
	Positive regulation of immune effector process	CLEC7A/HLA-DMB/HLA-DRA/HLA-DRB1/IL6/ITGAM/ITGB2/MYB/NOD2/PCK1/PLA2G5/RAC2/RARA/SLAMF6/SLC7A5/TNF/TYROBP/WNT5A	18	6.62E-09
	Leukocyte cell-cell adhesion	AIF1/CD70/EGR3/FOXJ1/HES1/HLA-DMB/HLA-DRA/HLA-DRB1/IL6/IRF1/ITGB2/LILRB4/MIR27A/MYB/NOD2/PAK3/PCK1/RAC2/RARA/S100A8/THY1/TNF/VSIG4	23	9.26E-09
	Reactive oxygen species metabolic process	AGTR2/CDKN1A/CLEC7A/CYBB/DDIT4/HBA1/HBA2/HBB/HBD/HBZ/HK2/ITGAM/ITGB2/MT3/PLIN5/PTGS2/RAC2/SESN2/TNF/TYROBP	20	1.12E-08
	Regulation of cell-cell adhesion	AIF1/CD70/EGR3/FOXJ1/HES1/HLA-DMB/HLA-DRA/HLA-DRB1/IL6/IRF1/ITGB2/LILRB4/MIR27A/MYB/NOD2/PAK3/PCK1/RARA/THY1/TNF/TNR/VEGFA/VSIG4/WNT3A/WNT5A	25	1.35E-08
	Response to glucocorticoid	ADM/AGTR2/ALPL/AVPR1A/CDKN1A/DDIT4/ERRFI1/FOSB/GJB2/HSD11B2/IL6/PCK1/STC1/TNF	14	1.75E-08
	Microglial cell activation	AIF1/C1QA/IL6/ITGAM/ITGB2/NR1D1/TLR8/TNF/TYROBP	9	2.98E-08
IRGs	Macrophage activation	AIF1/ITGAM/ITGB2/TYROBP/VSIG4/TLR8	6	2.61349E-10
	Microglial cell activation	AIF1/ITGAM/ITGB2/TYROBP/TLR8	5	7.57363E-10
	Leukocyte activation involved in inflammatory response	AIF1/ITGAM/ITGB2/TYROBP/TLR8	5	7.57363E-10
	Glial cell activation	AIF1/ITGAM/ITGB2/TYROBP/TLR8	5	2.01495E-09
	Positive regulation of superoxide anion generation	ITGAM/ITGB2/TYROBP/CLEC7A	4	2.32257E-09
	Regulation of superoxide anion generation	ITGAM/ITGB2/TYROBP/CLEC7A	4	4.37539E-09
	Neuroinflammatory response	AIF1/ITGAM/ITGB2/TYROBP/TLR8	5	7.51501E-09
	Superoxide anion generation	ITGAM/ITGB2/TYROBP/CLEC7A	4	3.49384E-08
	Regulation of superoxide metabolic process	ITGAM/ITGB2/TYROBP/CLEC7A	4	3.49384E-08
	Positive regulation of reactive oxygen species metabolic process	AIF1/ITGAM/ITGB2/TYROBP/CLEC7A	5	3.57011E-08

**Abbreviations:** DEGs: Differentially expressed genes, IRGs: Immune-related genes. “Count” means how many genes are involved.

**Table 3:** Top 10 Pathway analyses results of DEGs and IRGs sorted according to adjusted *P*-value.

**Group**	**Term**	**Gene**	**Count**	***P*-value**
DEGs	Leishmaniasis	CYBB/FCGR3A/HLA-DMB/HLA-DRA/HLA-DRB1/HLA-DRB5/ITGAM/ITGB2/JUN/PTGS2/TNF	11	2.94E-07
	Staphylococcus aureus infection	C1QA/C1QB/C1QC/FCGR3A/HLA-DMB/HLA-DRA/HLA-DRB1/HLA-DRB5/CFI/ITGAM/ITGB2	11	2.80E-06
	Hematopoietic cell lineage	ANPEP/HLA-DMB/HLA-DRA/HLA-DRB1/HLA-DRB5/IL6/IL11/ITGAM/KIT/MME/TNF	11	3.80E-06
	Rheumatoid arthritis	HLA-DMB/HLA-DRA/HLA-DRB1/HLA-DRB5/IL6/IL11/ITGB2/JUN/TNF/VEGFA	10	1.43E-05
	Pertussis	C1QA/C1QB/C1QC/IL6/IRF1/ITGAM/ITGB2/JUN/TNF	9	1.77E-05
	Inflammatory bowel disease	HLA-DMB/HLA-DRA/HLA-DRB1/HLA-DRB5/IL6/JUN/TNF/NOD2	8	3.95E-05
	Complement and coagulation cascades	C1QA/C1QB/C1QC/C9/CFI/ITGAM/ITGB2/F2RL3/VSIG4	9	4.39E-05
	Phagosome	CYBB/FCGR3A/HLA-DMB/HLA-DRA/HLA-DRB1/HLA-DRB5/ITGAM/ITGB2/MSR1/MARCO/TUBB3/CLEC7A	12	4.65E-05
	TNF signaling pathway	IL6/IRF1/JUN/LIF/MMP9/PTGS2/TNF/TNFRSF1B/SOCS3/NOD2	10	7.23E-05
	IL-17 signaling pathway	FOSB/IL6/JUN/MMP9/MMP13/PTGS2/S100A8/TNF/MAPK15	9	9.73E-05
IRGs	Staphylococcus aureus infection	C1QB/C1QC/HLA-DMB/HLA-DRB1/ITGAM/ITGB2	6	1.75344E-08
	Phagosome	HLA-DMB/HLA-DRB1/ITGAM/ITGB2/MARCO/CLEC7A	6	2.76073E-07
	Complement and coagulation cascades	C1QB/C1QC/ITGAM/ITGB2/VSIG4	5	4.57422E-07
	Pertussis	C1QB/C1QC/ITGAM/ITGB2	4	1.20992E-05
	Leishmaniasis	HLA-DMB/HLA-DRB1/ITGAM/ITGB2	4	1.2747E-05
	Tuberculosis	HLA-DMB/HLA-DRB1/ITGAM/ITGB2/CLEC7A	5	1.86187E-05
	Systemic lupus erythematosus	C1QB/C1QC/HLA-DMB/HLA-DRB1	4	0.000119669
	Cell adhesion molecules	HLA-DMB/HLA-DRB1/ITGAM/ITGB2	4	0.000170414
	Viral myocarditis	HLA-DMB/HLA-DRB1/ITGB2	3	0.000203827
	Antigen processing and presentation	HLA-DMB/HLA-DRB1/IFI30	3	0.000443376

**Abbreviations:** DEGs: Differentially expressed genes, IRGs: Immune-related genes. “Count” means how many genes are involved.

## Data Availability

The data supporting the findings of this study are available within the article.

## LIST OF ABBREVIATIONS

OA: Osteoarthritis
GEO: Gene Expression Omnibus
DEGs: Differentially Expressed Genes
IRGs: Immune-Related Genes
GO: Gene Ontology
KEGG: Kyoto Encyclopedia of Gene and Genomes
PPI: Protein-Protein Interaction
TF: Transcription Factor
ROC: Receiver Operating Characteristics
AUC: Area Under the Curve
