# Supplementary material for: Identification of Key Diagnostic Markers and Immune Infiltration in Osteoarthritis
Source: Comb Chem High Throughput Screen. 2023 Jan 5;26(2):410–23. doi: 10.2174/1386207325666220426083526 (PMC10202085; doi:10.2174/1386207325666220426083526)
Supplement: Supplementary file 1 [file CCHTS-26-410_SD1.pdf]

Supplementary Material

Identification of Key Diagnostic Markers and Immune Infiltration in Osteoarthritis

Mingyue Yan[1], Haibo Zhao[1], Zewen Sun[1], Jinli Chen[1], Yi Zhang[1], Jiake Gao[1] and Tengbo Yu[1]

[1]*Department of Orthopaedics, The Affiliated Hospital of Qingdao University, 39 HAIER ROAD, Qingdao, Shandong, China, 266500*

Table S1.

	baseMean	log2FoldChange	lfcSE	stat	pvalue	padj	sig
ABCA13	8.017568954	2.095266018	0.781354554	2.681581632	0.007327503	0.039039977	Up
ACPP	7.542639585	-2.130261449	0.701957357	-3.034744816	0.002407393	0.016692066	Down
ADAMTS14	44.21528949	2.012836699	0.417165409	4.825032598	1.40E-06	3.80E-05	Up
ADAMTSL5	4.19435423	2.227909802	0.550458583	4.047370452	5.18E-05	0.000785957	Up
ADM	1473.179919	-3.895628695	0.318335362	-12.23749905	1.96E-34	8.76E-31	Down
AGTR2	2.566413487	3.619410244	1.019402819	3.550520143	0.000384471	0.004021307	Up
AIF1	20.22321021	2.518797708	0.546756612	4.606798811	4.09E-06	9.48E-05	Up
AKR7A2P1	22.45136356	-2.289074661	0.50410611	-4.54085879	5.60E-06	0.000122577	Down
ALDH1L1	124.026594	-2.285908287	0.297908689	-7.67318434	1.68E-14	3.70E-12	Down
ALPL	50.42055706	2.855686378	0.699035661	4.085179825	4.40E-05	0.000687566	Up
AMTN	146.9067082	4.287475116	0.638849375	6.711245697	1.93E-11	2.14E-09	Up
ANGPTL4	1710.543837	-2.667756232	0.299794511	-8.898615994	5.65E-19	3.06E-16	Down
ANLN	40.83201701	2.374635304	0.436791897	5.436536989	5.43E-08	2.33E-06	Up
ANPEP	193.8808791	2.356888511	0.456749378	5.160135134	2.47E-07	8.82E-06	Up
APOBEC3B	2.042202642	2.590977897	0.880714977	2.941902847	0.003262022	0.021004558	Up
ASPM	43.53292345	3.269043946	0.491634526	6.649337612	2.94E-11	3.13E-09	Up
ASTL	2.987623398	-3.065111517	0.683673041	-4.483300249	7.35E-06	0.000155291	Down
ATF3	143.5858966	-3.501403443	0.340001053	-10.29821352	7.18E-25	9.17E-22	Down
ATOH8	509.000852	-2.104029846	0.280910484	-7.490036738	6.89E-14	1.34E-11	Down
AVPR1A	67.85417358	-2.424660513	0.456640609	-5.309778558	1.10E-07	4.34E-06	Down
BASP1	87.05813526	2.283427246	0.448295378	5.093577489	3.51E-07	1.18E-05	Up
BHLHE40	5158.621779	-2.05526357	0.230700179	-8.908807872	5.16E-19	2.88E-16	Down
BIRC5	13.75615542	2.10229565	0.612509362	3.432266967	0.000598558	0.005657972	Up
BIRC7	9.626902255	2.192365018	0.462151548	4.743822734	2.10E-06	5.36E-05	Up
BRE-AS1	16.66389014	-2.098852856	0.329888963	-6.362300928	1.99E-10	1.72E-08	Down
BTG2	1082.421214	-2.164804916	0.311710629	-6.944918498	3.79E-12	5.01E-10	Down

BUB1	11.61912276	2.072725167	0.48335579	4.288197654	1.80E-05	0.00032956	Up
BUB1B	7.953368803	2.304188039	0.695872154	3.311223229	0.000928891	0.007929285	Up
C16orf54	4.429341497	3.129247809	0.669688549	4.672691226	2.97E-06	7.28E-05	Up
C19orf35	27.66107613	-2.513787659	0.303271418	-8.288903955	1.14E-16	3.93E-14	Down
C1QA	38.87513455	2.361913885	0.526375994	4.487123106	7.22E-06	0.000152893	Up
C1QB	84.07334008	3.024433351	0.602937483	5.016164087	5.27E-07	1.68E-05	Up
C1QC	47.81069132	2.196330607	0.559359647	3.926508858	8.62E-05	0.001208318	Up
C1QL1	3.220579893	3.038652033	0.742090174	4.094720746	4.23E-05	0.000666846	Up
C1orf100	1.157699412	2.024763658	0.673126184	3.008000144	0.00262973	0.017825721	Up
C1orf233	6.452629597	2.73411592	0.649004055	4.212787116	2.52E-05	0.000436896	Up
C1orf87	11.67386323	-2.017747931	0.559342974	-3.607353668	0.000309336	0.003400602	Down
C2CD4B	5.131082298	-2.713076425	0.754924748	-3.593836914	0.000325844	0.003540704	Down
C6orf223	6.106460846	-2.482335221	0.522756247	-4.748551994	2.05E-06	5.25E-05	Down
C9	3.737084008	2.375798417	0.558091487	4.257005295	2.07E-05	0.000371455	Up
CBLN4	3.643634778	2.669253897	0.799286761	3.339544739	0.000839158	0.007343251	Up
CCKAR	2.759049454	3.282056195	0.794338865	4.13180865	3.60E-05	0.000580647	Up
CCL4	4.311565213	2.985454354	0.851092863	3.507789202	0.000451847	0.004552855	Up
CCR1	11.12879509	2.029998778	0.525345957	3.864118019	0.000111491	0.001479515	Up
CCR5	3.986644323	2.415915482	0.861430079	2.804540427	0.005038837	0.029319404	Up
CD163	77.12996856	2.206011515	0.564283911	3.909399985	9.25E-05	0.001273207	Up
CD300C	14.60666864	2.451500719	0.393000232	6.23791163	4.43E-10	3.45E-08	Up
CD48	3.115880424	2.514617079	0.734369569	3.424184749	0.000616647	0.005803279	Up
CD69	1.94294093	2.594252997	0.931541695	2.784902717	0.005354378	0.030685962	Up
CD70	4.461487935	3.638860865	0.835446695	4.355587123	1.33E-05	0.000256454	Up
CDH10	5.082556472	3.060233888	0.667845106	4.582250977	4.60E-06	0.00010395	Up
CDH2	13.42990348	2.964230196	0.549781545	5.391650964	6.98E-08	2.92E-06	Up
CDHR4	21.13409998	2.030862824	0.328226751	6.187377534	6.12E-10	4.56E-08	Up
CDK1	13.75249321	2.046750211	0.504097124	4.060229889	4.90E-05	0.00075091	Up
CDKN1A	554.0726654	-2.689867065	0.274378462	-9.803492037	1.09E-22	8.45E-20	Down
CENPF	57.95822557	2.162131963	0.416519288	5.190952801	2.09E-07	7.66E-06	Up
CEP55	8.036367999	2.377279034	0.664653755	3.576717975	0.000347935	0.003728188	Up
CFI	190.5502614	2.744015532	0.272541017	10.06826627	7.63E-24	7.18E-21	Up
CHI3L2	12398.63573	2.090294873	0.476336077	4.388277465	1.14E-05	0.000224918	Up
CHST4	2.37652207	2.030024606	0.656151545	3.093834985	0.001975874	0.014404053	Up
CISH	175.4711697	-2.810499422	0.259315838	-10.83813254	2.27E-27	5.80E-24	Down
CLDN5	48.08128081	-2.43891384	0.390910327	-6.239062187	4.40E-10	3.44E-08	Down
CLEC12A	5.538318102	2.733849223	0.641012334	4.264893325	2.00E-05	0.000361838	Up
CLEC12B	1.39457087	3.59446997	1.174201441	3.061203849	0.00220449	0.015604435	Up
CLEC7A	11.65352119	2.205598691	0.500299114	4.408560061	1.04E-05	0.000207598	Up

COL1A1	11273.5738	4.26779308	0.58434276	7.303578257	2.80E-13	4.77E-11	Up
COL1A2	20247.3152	2.287867784	0.393739518	5.810612543	6.22E-09	3.34E-07	Up
CPA4	2.186845489	2.312772953	0.891839998	2.593259954	0.009507088	0.047588686	Up
CPN2	2.626470194	2.379342722	0.657893611	3.616607128	0.00029849	0.003306684	Up
CPSF4L	1.36844808	-2.132489642	0.675033903	-3.159085244	0.001582652	0.012069071	Down
CPZ	21.122869	2.324148117	0.447843946	5.189638345	2.11E-07	7.70E-06	Up
CSN1S1	23.85905451	4.806296621	0.579568581	8.292886782	1.11E-16	3.87E-14	Up
CSRNP1	520.8908383	-2.917588198	0.318843058	-9.150546393	5.66E-20	3.62E-17	Down
CSTA	1.886057536	2.932835335	0.951378923	3.082720526	0.002051177	0.014799193	Up
CXCL14	156.6543524	2.298763175	0.562098528	4.089608957	4.32E-05	0.000678122	Up
CYBB	50.7904079	2.176454692	0.515818413	4.21942032	2.45E-05	0.00042589	Up
CYP4B1	9.846277538	-3.869671691	0.980062392	-3.948393209	7.87E-05	0.001116159	Down
CYTIP	5.130358791	2.196678324	0.617703741	3.556200452	0.000376257	0.003949263	Up
DCDC2	1.222201151	3.39996253	1.317367519	2.580876241	0.00985499	0.048662418	Up
DDIT3	268.9968967	-2.376066576	0.176246314	-13.48151074	2.01E-41	1.80E-37	Down
DDIT4	5458.631224	-3.3096081	0.321349903	-10.29907919	7.11E-25	9.17E-22	Down
DHRS13	73.0144946	-2.209206773	0.215039517	-10.27349207	9.28E-25	1.11E-21	Down
DIAPH3	15.04150551	2.361956788	0.607969568	3.884991802	0.000102333	0.001383668	Up
DIO2	670.7444931	2.295762544	0.359963055	6.37777269	1.80E-10	1.57E-08	Up
DMBT1	12.15554836	2.318088553	0.407638968	5.686621588	1.30E-08	6.52E-07	Up
DPYS	2.317547378	2.432722939	0.825103908	2.948383733	0.003194403	0.020738558	Up
DUSP2	23.85558483	-3.621975634	0.442009565	-8.194337672	2.52E-16	7.63E-14	Down
EGR3	130.5211681	-2.079943157	0.327589938	-6.349227848	2.16E-10	1.80E-08	Down
ELF3	46.11543752	-3.077145987	0.383870012	-8.016114546	1.09E-15	2.99E-13	Down
EN1	7.063871902	2.162568344	0.576175283	3.753316758	0.00017451	0.002132172	Up
ENAM	3.492292673	2.211861951	0.841551893	2.628313202	0.008580947	0.044063323	Up
EPDR1	51.83942587	2.076759202	0.265267958	7.828910882	4.92E-15	1.16E-12	Up
ERRFI1	5728.67326	-2.054014465	0.2458037	-8.356320364	6.47E-17	2.41E-14	Down
F2RL3	31.89426766	-2.228545416	0.350498207	-6.35822202	2.04E-10	1.74E-08	Down
FAM135B	5.995825111	2.038301702	0.656432921	3.105118035	0.001902031	0.013997038	Up
FAM166A	18.19985577	-2.767904644	0.311943444	-8.873097675	7.11E-19	3.63E-16	Down
FAM43A	169.9367692	-2.19183894	0.277700899	-7.892804628	2.95E-15	7.23E-13	Down
FAM71A	2.799747831	-2.534310911	0.712187583	-3.558487922	0.000372996	0.003917334	Down
FCGR3A	23.67027625	2.393580932	0.460206443	5.201102611	1.98E-07	7.30E-06	Up
FCRL5	2.706267719	3.23734836	1.11030657	2.915724763	0.003548634	0.022335152	Up
FGF9	6.065173079	2.693155265	0.53759003	5.009682316	5.45E-07	1.73E-05	Up
FOSB	2176.409965	-3.423280841	0.467538492	-7.321922999	2.44E-13	4.24E-11	Down
FOSL2	1172.163337	-2.170644897	0.247267785	-8.778518785	1.66E-18	7.59E-16	Down
FOXJ1	1.615564317	-2.834222397	0.903760423	-3.136032872	0.0017125	0.012840159	Down

G0S2	180.8031822	-2.558185257	0.423686235	-6.037923932	1.56E-09	1.05E-07	Down
GABRB2	12.54916132	2.026540252	0.702075286	2.886499912	0.003895529	0.024036097	Up
GADD45B	1349.295328	-2.535726252	0.250811148	-10.11010186	4.98E-24	4.95E-21	Down
GALR3	1.119736835	-2.849999389	0.757372409	-3.763009259	0.000167881	0.002069567	Down
GDNF	2.019325021	2.672355916	0.82074726	3.256003457	0.001129924	0.009218342	Up
GFRA2	94.68368413	2.06678622	0.34948321	5.913835521	3.34E-09	2.00E-07	Up
GJB2	31.23017474	3.370189197	0.472214252	7.136991692	9.54E-13	1.42E-10	Up
GLYAT	1.319030102	2.509053824	0.971174328	2.583525689	0.009779618	0.048491173	Up
GMNC	1.250257036	3.124305394	1.202921095	2.597265447	0.009396927	0.047116431	Up
GRIA2	61.2586703	3.885035093	0.48492512	8.011618571	1.13E-15	3.02E-13	Up
HBA1	4.794152275	2.283883884	0.54095436	4.221953003	2.42E-05	0.000422549	Up
HBA2	14.84941095	2.890822797	0.528363348	5.47127807	4.47E-08	1.97E-06	Up
HBB	143.1890398	4.851488873	0.666069689	7.283755668	3.25E-13	5.37E-11	Up
HBD	1.825002569	4.036862653	1.040923312	3.878155677	0.000105251	0.00141776	Up
HBZ	1.286464646	-2.452033227	0.800634374	-3.06261298	0.002194136	0.01554506	Down
HCN1	3.200734606	4.389582712	1.041227715	4.215775907	2.49E-05	0.000431567	Up
HCN2	1.223825058	2.30727395	0.778286375	2.964556523	0.003031196	0.019942076	Up
HERC5	89.33570326	-2.058498366	0.272097118	-7.565307504	3.87E-14	7.86E-12	Down
HES1	816.6028874	-2.079817913	0.283111526	-7.346284842	2.04E-13	3.57E-11	Down
HILPDA	1406.064613	-3.473509944	0.277143307	-12.53326296	4.91E-36	2.93E-32	Down
HIST1H1D	9.469821385	-3.449559563	0.700665898	-4.92325882	8.51E-07	2.48E-05	Down
HIST1H3B	4.41524816	2.453043151	0.91782608	2.672666645	0.007525099	0.039786943	Up
HJURP	8.757066198	2.00973026	0.603546266	3.329869428	0.000868867	0.007513789	Up
HK2	737.9486929	-2.186895992	0.246463233	-8.873112492	7.11E-19	3.63E-16	Down
HLA-DMB	18.9146665	2.099858586	0.525368431	3.996925702	6.42E-05	0.000941746	Up
HLA-DRA	180.457467	2.79365718	0.604860149	4.618682821	3.86E-06	9.01E-05	Up
HLA-DRB1	110.4917991	2.577341937	0.555044062	4.643490698	3.43E-06	8.22E-05	Up
HLA-DRB5	25.53540135	2.220630807	0.566130809	3.922469455	8.76E-05	0.001224629	Up
HMGA2	17.46910222	2.470039054	0.393277157	6.280657314	3.37E-10	2.69E-08	Up
HPGDS	8.572019535	3.141486662	0.62582668	5.019739113	5.17E-07	1.66E-05	Up
HPN	10.65697697	-2.52989901	0.50227214	-5.036908896	4.73E-07	1.53E-05	Down
HSD11B2	15.92520219	-2.072459652	0.465652173	-4.450660323	8.56E-06	0.00017609	Down
HTRA1	16494.84128	2.25060789	0.267974437	8.398591736	4.52E-17	1.76E-14	Up
IER2	704.9453351	-2.315420006	0.235116467	-9.84797039	6.99E-23	5.95E-20	Down
IFI30	116.6145977	2.043517639	0.374611894	5.455026053	4.90E-08	2.13E-06	Up
IFNA22P	1.945937192	2.027476531	0.730897745	2.773953737	0.005537955	0.031545873	Up
IFNA5	2.566979916	2.362681642	0.648069349	3.645723482	0.000266641	0.003008965	Up
IFNE	7.398609446	2.493313956	0.46908978	5.315216963	1.07E-07	4.23E-06	Up
IGFBP1	13.79125525	6.542762771	0.849257986	7.704093312	1.32E-14	2.94E-12	Up

IGFBP3	178.5529896	2.394538411	0.428567762	5.5873041	2.31E-08	1.08E-06	Up
IGFL2	2.420270316	2.256938044	0.699547502	3.226282756	0.001254094	0.010012027	Up
IL11	18.11584404	3.371164154	0.86136148	3.913762377	9.09E-05	0.001256214	Up
IL6	5.905608718	-2.972390443	0.714437702	-4.160461344	3.18E-05	0.000521322	Down
IRF1	234.3205694	-2.344084296	0.339346007	-6.907652508	4.93E-12	6.20E-10	Down
IRF4	22.49879813	-2.018007898	0.47017656	-4.292021488	1.77E-05	0.000325936	Down
ITGAM	36.93234239	2.24636405	0.435656333	5.156275442	2.52E-07	8.97E-06	Up
ITGB2	54.29619957	2.559912659	0.493413109	5.188173176	2.12E-07	7.75E-06	Up
ITPRIP	560.6465273	-2.108050577	0.263022701	-8.014709638	1.10E-15	2.99E-13	Down
JUN	1629.653468	-2.760349786	0.256491624	-10.76194904	5.21E-27	9.72E-24	Down
KCNJ1	2.401118714	2.008420217	0.648741096	3.09587327	0.001962342	0.01433464	Up
KCNJ9	9.393898726	-2.018810584	0.380679089	-5.303182234	1.14E-07	4.49E-06	Down
KCNN4	71.51222632	2.104010764	0.357363991	5.887584688	3.92E-09	2.29E-07	Up
KCNS3	18.1191026	2.162801805	0.378753963	5.710308058	1.13E-08	5.74E-07	Up
KIAA1683	119.3740702	-2.054612448	0.307443966	-6.682884273	2.34E-11	2.57E-09	Down
KIF23	14.08218936	2.195858908	0.478829729	4.585886748	4.52E-06	0.000102676	Up
KIT	71.84283473	-2.962812436	0.396665175	-7.469303142	8.06E-14	1.55E-11	Down
KLF10	886.2500691	-2.016740109	0.173315043	-11.63626694	2.70E-31	8.03E-28	Down
KLHL4	3.504892032	2.897882144	0.852691019	3.398513739	0.000677531	0.006268561	Up
LDHC	7.842671478	-2.136992063	0.492306518	-4.340775485	1.42E-05	0.000271305	Down
LGALS8-AS1	5.165276868	-2.24162253	0.553807002	-4.047660142	5.17E-05	0.000785957	Down
LIF	66.47408374	-2.198528443	0.550634012	-3.992721833	6.53E-05	0.000953703	Down
LILRB4	10.89943929	2.796758623	0.528822938	5.288648475	1.23E-07	4.79E-06	Up
LILRB5	20.35848169	2.386090006	0.510559392	4.673481764	2.96E-06	7.26E-05	Up
LINC00520	1.763549602	2.746231645	0.857990096	3.200773129	0.001370594	0.010735919	Up
LINC00671	2.518214414	2.626817661	0.731686991	3.590083868	0.000330572	0.003583364	Up
LINGO3	7.376583954	-2.547014881	0.459303371	-5.545386866	2.93E-08	1.35E-06	Down
LIPF	1.543689075	3.088752413	1.127408583	2.739692122	0.006149676	0.034164425	Up
LNX1-AS1	3.376147732	2.478223926	0.694769448	3.566973091	0.000361129	0.003828691	Up
LOC100130264	1.088490164	2.424030393	0.943392363	2.569482739	0.010185047	0.04976974	Up
LOC100132077	4.58751178	2.053831533	0.471391304	4.35695677	1.32E-05	0.000255408	Up
LOC100506801	13.86423073	-2.039478537	0.343944777	-5.929668586	3.04E-09	1.84E-07	Down
LOC100507472	2.47483464	3.192230074	0.733986389	4.349167943	1.37E-05	0.000262657	Up
LOC143666	92.04867494	-2.818911875	0.267983897	-10.51895991	7.06E-26	1.15E-22	Down
LOC399715	79.31780846	-2.347248029	0.292247589	-8.031710489	9.61E-16	2.68E-13	Down
LOC400940	12.74718078	2.90826826	0.436239442	6.666678851	2.62E-11	2.82E-09	Up
LOC728084	2.436210658	2.08388092	0.754306232	2.762645769	0.005733495	0.032452891	Up
LOC729966	1.961613454	-2.995269265	0.953940974	-3.139889517	0.001690116	0.012704296	Down
LONRF3	78.56662456	-2.041750444	0.299082753	-6.826707405	8.69E-12	1.04E-09	Down

LPAL2	9.214595609	2.140218211	0.391788963	5.462681225	4.69E-08	2.05E-06	Up
LRRC15	246.04529	3.748644142	0.489643007	7.655871913	1.92E-14	4.19E-12	Up
LY6D	6.168005386	2.765899564	0.594276495	4.654230122	3.25E-06	7.89E-05	Up
LYZ	63.05044602	2.567133404	0.458144961	5.603321268	2.10E-08	1.01E-06	Up
MAB21L3	13.15423394	-2.035523654	0.438794091	-4.638903981	3.50E-06	8.35E-05	Down
MAFF	265.6633187	-2.986674816	0.177887057	-16.78972526	2.90E-63	5.19E-59	Down
MAGI2-AS2	1.369866793	2.230446876	0.798134778	2.794574221	0.005196811	0.030052528	Up
MAOA	84.54040562	-2.172300808	0.422868464	-5.137060323	2.79E-07	9.80E-06	Down
MAPK15	6.704117197	2.364302052	0.564469431	4.18853869	2.81E-05	0.000475239	Up
MARCO	47.27942909	2.90344416	0.474227481	6.122471336	9.21E-10	6.63E-08	Up
MIR1245A	4.592017119	2.934156751	0.750624861	3.908952263	9.27E-05	0.001274588	Up
MIR1245B	4.763793702	3.222030816	0.748277524	4.305930234	1.66E-05	0.000310266	Up
MIR210	2.492669499	-2.414759831	0.564425873	-4.278258573	1.88E-05	0.000343918	Down
MIR23A	6.609983748	-2.562281244	0.608488342	-4.210896196	2.54E-05	0.000439717	Down
MIR24-2	3.073335634	-2.241772835	0.608855403	-3.681946199	0.00023146	0.002674436	Down
MIR27A	4.420044951	-2.355717706	0.63644375	-3.701376135	0.000214433	0.002521488	Down
MIR3190	1.687499217	-2.454423944	0.845277503	-2.903690132	0.00368793	0.022961248	Down
MIR3606	3.574538779	2.498480267	0.677302324	3.688870065	0.000225252	0.002621343	Up
MIR3609	4.006132331	-2.419884462	0.926467601	-2.611947206	0.009002816	0.045587912	Down
MIR4308	3.171277466	-2.901977882	0.739640463	-3.923498006	8.73E-05	0.001222567	Down
MIR4505	5.297795375	-3.462844817	0.593805516	-5.83161443	5.49E-09	3.01E-07	Down
MIR4530	1.421713294	-2.07051203	0.680573616	-3.042304289	0.002347744	0.016373753	Down
MIR5087	5.517612308	-3.908963668	0.707107879	-5.528100849	3.24E-08	1.47E-06	Down
MIR548O2	1.63067328	-2.644328143	0.861745465	-3.068572159	0.002150844	0.015317264	Down
MIR614	6.852371419	-2.368840584	0.577284534	-4.103419448	4.07E-05	0.000643956	Down
MKI67	58.20838514	2.798283333	0.520127821	5.379991645	7.45E-08	3.10E-06	Up
MME	20.91010694	2.383030542	0.620317643	3.841629478	0.00012222	0.001596993	Up
MMP11	19.64464196	2.236897832	0.548549244	4.077843251	4.55E-05	0.000706535	Up
MMP13	126.2029682	2.670837842	0.478540901	5.581211209	2.39E-08	1.12E-06	Up
MMP19	53.58470713	2.232128127	0.40785742	5.4728148	4.43E-08	1.96E-06	Up
MMP9	41.21184529	2.40863592	0.699650924	3.442625226	0.000576097	0.005500927	Up
MSMP	254.9016991	-2.428519355	0.589123328	-4.122259706	3.75E-05	0.000601456	Down
MSR1	35.85542882	2.241218409	0.501891481	4.46554384	7.99E-06	0.000166775	Up
MT3	4.427765693	-2.414936675	0.878303458	-2.74954704	0.00596777	0.033325174	Down
MTFP1	69.60896561	-2.162162044	0.212398483	-10.17974334	2.44E-24	2.57E-21	Down
MYB	2.157226761	3.047650707	0.924785065	3.295523276	0.000982386	0.008290908	Up
MYO7A	124.1266557	-2.430160672	0.272419309	-8.920662334	4.64E-19	2.67E-16	Down
NAT2	8.039876138	2.184129479	0.403628945	5.411231043	6.26E-08	2.66E-06	Up
NDST3	2.872355763	2.542963152	0.949286745	2.678814557	0.00738833	0.039293781	Up

NELL1	19.36290531	3.159939463	0.630384228	5.012719737	5.37E-07	1.71E-05	Up
NFIL3	409.7757458	-2.511254095	0.321135848	-7.819912064	5.29E-15	1.23E-12	Down
NGF	39.64393943	2.78180926	0.563889736	4.933250385	8.09E-07	2.37E-05	Up
NKAIN4	1.678334958	3.381595484	0.924867062	3.656304375	0.000255878	0.002911747	Up
NKD2	2.328266911	2.584327598	0.798496136	3.236493555	0.00121008	0.009725797	Up
NOD2	47.96531293	-2.171982144	0.327887368	-6.624171456	3.49E-11	3.63E-09	Down
NOV	53.49571515	2.522068933	0.318771291	7.911844657	2.54E-15	6.38E-13	Up
NPTX2	15.31622621	2.529610315	0.474369311	5.332575812	9.68E-08	3.95E-06	Up
NR1D1	2005.343574	-2.18345227	0.384711634	-5.675555598	1.38E-08	6.88E-07	Down
NUF2	6.484029833	2.68973072	0.612298914	4.392839281	1.12E-05	0.000220734	Up
ODAM	1.885726901	3.453344317	1.058234076	3.263308558	0.001101196	0.009045896	Up
OGN	9280.196532	2.242161635	0.321023219	6.984421999	2.86E-12	3.82E-10	Up
OPRK1	7.896497961	-2.73076569	0.760114273	-3.592572576	0.000327429	0.003551457	Down
OPTC	19.54094119	-2.380168912	0.322969065	-7.369649828	1.71E-13	3.03E-11	Down
PAK3	15.00228822	-2.100226666	0.374158161	-5.613205545	1.99E-08	9.62E-07	Down
PAQR4	18.06310058	2.070222312	0.425139639	4.869511382	1.12E-06	3.14E-05	Up
PARP15	12.13175373	2.257938754	0.457984618	4.930162856	8.22E-07	2.40E-05	Up
PAX1	7.465112165	2.210755988	0.657396443	3.362896182	0.000771294	0.006880997	Up
PBK	9.180385252	2.466172622	0.64779453	3.807029091	0.000140646	0.001777971	Up
PCDH17	28.42222614	-2.64694742	0.410127828	-6.453957132	1.09E-10	1.00E-08	Down
PCDHAC1	1.386306228	2.249762901	0.735194898	3.060090471	0.002212701	0.01562704	Up
PCK1	67.71742037	-2.168746353	0.443501241	-4.890057007	1.01E-06	2.88E-05	Down
PENK	125.0157141	4.346050507	0.626784344	6.933884913	4.09E-12	5.23E-10	Up
PFKFB3	3519.590351	-2.199129242	0.180003291	-12.21716132	2.52E-34	9.00E-31	Down
PHACTR3	3.387209807	2.953557397	0.555958629	5.312548886	1.08E-07	4.28E-06	Up
PIK3CG	7.342402361	2.171517468	0.581827292	3.732237209	0.000189787	0.002279863	Up
PLA2G5	3.091735098	3.205187445	0.89893895	3.565522936	0.000363131	0.003840173	Up
PLEKHS1	5.653162696	3.215007659	0.632696165	5.081440089	3.75E-07	1.25E-05	Up
PLIN5	13.84948869	-2.755105918	0.663195553	-4.154288888	3.26E-05	0.000534613	Down
PLK2	51.21137508	-2.449373657	0.371256824	-6.597518217	4.18E-11	4.22E-09	Down
POSTN	1567.505206	4.498943314	0.703833099	6.392059878	1.64E-10	1.45E-08	Up
PPP1R14C	82.88792464	2.261215265	0.33008422	6.850419156	7.36E-12	9.08E-10	Up
PPP1R15A	485.1467951	-2.02586812	0.221905118	-9.12943396	6.89E-20	4.24E-17	Down
PPP1R27	1.819994328	-2.621957775	0.809772518	-3.237894243	0.001204154	0.009682528	Down
PRAME	3.160290305	2.63766217	0.941310781	2.802116179	0.005076859	0.029463912	Up
PREX2	99.32052209	2.181212556	0.324101376	6.730031776	1.70E-11	1.91E-09	Up
PROZ	2.114283754	2.490587837	0.589290073	4.226420826	2.37E-05	0.000416098	Up
PRSS23	314.0939196	2.060036793	0.343063538	6.004825829	1.92E-09	1.24E-07	Up
PTGS2	209.5192599	-2.242920021	0.338570991	-6.624666844	3.48E-11	3.63E-09	Down

RAB27B	5.566418767	2.340471172	0.518537884	4.513597263	6.37E-06	0.000136935	Up
RAC2	9.466945053	2.769622119	0.481826002	5.74817903	9.02E-09	4.67E-07	Up
RARA	753.4343848	-2.039064352	0.300920054	-6.776099918	1.23E-11	1.43E-09	Down
RGAG1	1.685415769	2.270290177	0.790096086	2.873435546	0.004060339	0.024796229	Up
RGS4	6.239824642	2.147019261	0.551940919	3.889943989	0.000100267	0.00136088	Up
RNASE1	117.0096097	2.172829008	0.67264053	3.230297479	0.001236615	0.009890152	Up
RND1	64.58385829	-4.407390497	0.514537701	-8.56572898	1.07E-17	4.46E-15	Down
RNF39	18.88194984	-2.006292057	0.241277255	-8.315297085	9.15E-17	3.27E-14	Down
RNU12	39.19395621	-2.990596002	0.696842286	-4.291639675	1.77E-05	0.000326161	Down
RNU4ATAC	9.211156124	-3.025531122	0.486980426	-6.212839287	5.20E-10	3.97E-08	Down
RPSAP52	4.013784112	3.384947819	0.622489585	5.437758156	5.40E-08	2.32E-06	Up
RSPO2	342.7833111	2.399578663	0.410520091	5.845216146	5.06E-09	2.83E-07	Up
RUNX1-IT1	13.71477129	2.185154806	0.470998751	4.639406791	3.49E-06	8.34E-05	Up
RXFP4	13.57789351	-2.177583749	0.31876253	-6.831366739	8.41E-12	1.01E-09	Down
S100A4	949.5564011	2.096609594	0.327522789	6.401415919	1.54E-10	1.38E-08	Up
S100A8	22.2125683	3.956151881	0.63992045	6.182255749	6.32E-10	4.63E-08	Up
SAA1	31.12802715	-2.048005776	0.726497467	-2.819012962	0.004817157	0.0282132	Down
SAA2	7.065797457	-2.019674938	0.753567034	-2.680152989	0.007358852	0.039171971	Down
SAMSN1	4.610005206	2.043478967	0.640006234	3.192904785	0.001408494	0.010976839	Up
SESN2	151.0172577	-2.535371312	0.235369258	-10.77188812	4.67E-27	9.72E-24	Down
SEZ6L2	16.8809551	2.37969618	0.462837492	5.141537198	2.72E-07	9.59E-06	Up
SFRP4	50.59523773	2.250286372	0.578203188	3.89186089	9.95E-05	0.001353253	Up
SHANK2-AS1	1.176585567	2.808760068	0.896815417	3.131926608	0.001736633	0.012999293	Up
SHC4	86.91930407	2.200631999	0.393198428	5.596746684	2.18E-08	1.04E-06	Up
SIK1	196.4557213	-2.766708872	0.382722204	-7.229026287	4.86E-13	7.58E-11	Down
SLAMF6	2.663798402	3.806501663	1.248168676	3.049669276	0.002290935	0.016052709	Up
SLC35G5	1.680931253	2.187734781	0.647738429	3.377497278	0.000731487	0.006637221	Up
SLC38A3	292.5945719	-2.74920537	0.276065757	-9.958516421	2.31E-23	2.07E-20	Down
SLC4A1	4.978588734	2.680963357	0.800161789	3.3505266	0.000806581	0.007126855	Up
SLC6A15	17.96116275	-2.135283599	0.434868285	-4.910184703	9.10E-07	2.63E-05	Down
SLC7A5	221.9811543	-2.155778656	0.271464995	-7.941276746	2.00E-15	5.18E-13	Down
SLC7A5P1	4.374698177	-2.225581962	0.471635264	-4.718862498	2.37E-06	5.98E-05	Down
SLC8A1-AS1	2.275889748	2.721675558	0.66618187	4.085484278	4.40E-05	0.000687265	Up
SLCO1C1	3.609338753	2.385675508	0.796820373	2.993994116	0.002753513	0.018524289	Up
SLITRK6	3.974729794	3.135031445	0.746743889	4.198268629	2.69E-05	0.000459629	Up
SLURP1	1.221009289	2.84582296	1.095576554	2.597557377	0.009388943	0.047089608	Up
SNORA80B	2.728341833	-3.087056606	0.655967761	-4.706110251	2.52E-06	6.32E-05	Down
SNORD116-24	1.271735565	2.746749864	0.687384302	3.995944997	6.44E-05	0.000944103	Up
SNORD43	1.889809541	-2.305811276	0.7607025	-3.031160376	0.002436158	0.01683926	Down

SNORD63	4.984646643	-2.038290053	0.479680609	-4.249265066	2.14E-05	0.000382605	Down
SNORD74	3.162766196	-2.024767376	0.759704449	-2.665204053	0.007694162	0.040387771	Down
SNX10	19.76355171	2.695858111	0.435511051	6.190102654	6.01E-10	4.53E-08	Up
SOCS3	1417.853274	-2.273512159	0.327515599	-6.941691215	3.87E-12	5.09E-10	Down
SOWAHA	4.638426337	-2.118338449	0.49222539	-4.303594432	1.68E-05	0.000312254	Down
SOX11	49.80036016	2.326734906	0.508474547	4.57591225	4.74E-06	0.000106609	Up
SPOCK1	358.0957215	2.529685837	0.285548271	8.859047987	8.07E-19	4.01E-16	Up
ST6GAL2	23.67937184	2.947474496	0.565806704	5.209331162	1.90E-07	7.04E-06	Up
ST6GALNAC5	42.61096435	3.47161043	0.450592773	7.704540865	1.31E-14	2.94E-12	Up
STAC2	234.3861557	-2.038569504	0.305587705	-6.670980109	2.54E-11	2.75E-09	Down
STC1	17.17441975	-2.752734266	0.516037042	-5.334373388	9.59E-08	3.93E-06	Down
STC2	2307.750769	-2.377628879	0.326592302	-7.280113039	3.34E-13	5.42E-11	Down
STON1-GTF2A1L	3.177801039	-2.294980136	0.50267758	-4.565511233	4.98E-06	0.000111473	Down
SUCNR1	2.247278325	2.760802805	0.63603512	4.340645219	1.42E-05	0.000271305	Up
TBL1Y	4.536146995	-2.270164297	0.736430625	-3.08265873	0.002051603	0.014799193	Down
TCF24	1.425051586	2.362012308	0.799034728	2.956082164	0.003115741	0.020355945	Up
TGFBI	2897.662545	2.96384049	0.496148124	5.973700892	2.32E-09	1.45E-07	Up
THEMIS2	35.02411223	2.108470339	0.359710022	5.861583527	4.58E-09	2.61E-07	Up
THY1	458.5066463	2.955458815	0.477836037	6.185089829	6.21E-10	4.59E-08	Up
TLL1	32.41864504	2.431999228	0.443962982	5.477932454	4.30E-08	1.92E-06	Up
TLR8	4.001115884	2.193247374	0.742858896	2.952441421	0.003152719	0.02053	Up
TMEM119	65.09732128	3.009755413	0.52948345	5.68432387	1.31E-08	6.58E-07	Up
TMEM151A	5.113039539	2.136527724	0.41669465	5.127322193	2.94E-07	1.03E-05	Up
TMEM158	5.798568657	2.411051492	0.653387555	3.690078691	0.000224185	0.002612322	Up
TMEM178B	5.051731144	2.221865758	0.668933545	3.321504469	0.000895335	0.007694289	Up
TMEM215	1.584402185	3.082039521	1.17374899	2.625808028	0.008644356	0.04432526	Up
TMEM59L	10.34223379	2.476387446	0.487585079	5.078882744	3.80E-07	1.26E-05	Up
TMEM63C	4.598489802	2.439671924	0.783858241	3.112389199	0.001855797	0.013718927	Up
TNC	11331.75008	2.432340926	0.456938859	5.323121196	1.02E-07	4.13E-06	Up
TNF	3.631072877	2.761864872	0.879579635	3.139982741	0.001689578	0.012704296	Up
TNFAIP6	515.1847808	2.994518269	0.509476902	5.877633033	4.16E-09	2.40E-07	Up
TNFRSF1B	84.70130923	-2.031380865	0.380695738	-5.335969547	9.50E-08	3.91E-06	Down
TNFSF15	46.60889099	3.185633369	0.529803586	6.012857314	1.82E-09	1.19E-07	Up
TNR	5.155489491	2.949757532	0.73440552	4.016524183	5.91E-05	0.000884575	Up
TOB1	725.7709869	-2.325777776	0.281244066	-8.269606558	1.34E-16	4.37E-14	Down
TOP2A	60.51006648	2.858414438	0.522465246	5.471013546	4.47E-08	1.97E-06	Up
TPPP3	443.565221	2.494079145	0.426500638	5.847773544	4.98E-09	2.80E-07	Up
TREM1	116.8089048	3.169378871	0.449858897	7.045273292	1.85E-12	2.58E-10	Up
TRIM31	1.114745657	-2.270481087	0.703984623	-3.225185628	0.00125891	0.010041508	Down

TRPA1	4.533550221	2.128012029	0.604615711	3.51961087	0.00043218	0.004388151	Up
TTC9	60.74333338	2.365874589	0.300128945	7.882860443	3.20E-15	7.73E-13	Up
TUBB3	25.85072644	2.413611988	0.44334864	5.444049605	5.21E-08	2.24E-06	Up
TXNIP	6492.182684	-2.1477452	0.317621352	-6.761967316	1.36E-11	1.55E-09	Down
TXNRD3NB	1.629841319	2.148026074	0.635103988	3.382164359	0.000719171	0.006572179	Up
TYROBP	65.74903937	3.130930433	0.619525413	5.053756258	4.33E-07	1.41E-05	Up
VEGFA	5038.851806	-2.573554765	0.246994242	-10.41949295	2.02E-25	3.01E-22	Down
VSIG4	58.39876365	3.018388771	0.607445713	4.968985219	6.73E-07	2.04E-05	Up
VWA5B2	4.321589847	2.361839496	0.46393426	5.090892607	3.56E-07	1.20E-05	Up
WNT3A	2.012182276	-2.200671878	0.618006136	-3.560922378	0.000369554	0.003888042	Down
WNT5A	16.54458031	2.393484655	0.581548621	4.115708588	3.86E-05	0.00061439	Up
ZC2HC1B	1.337456455	2.029832082	0.697946942	2.908289957	0.003634112	0.022729095	Up
ZNF474	4.89961306	2.063853652	0.486346746	4.243584786	2.20E-05	0.000390476	Up
ZNF804A	1.687951658	2.512510567	0.897582561	2.799197171	0.005122984	0.029668174	Up
ZNF878	9.086780178	-2.477536991	0.558151497	-4.438825305	9.05E-06	0.000184357	Down
